# Family concerns in organ donor conversations: a qualitative embedded multiple-case study

**DOI:** 10.1186/s13054-024-05198-2

**Published:** 2024-12-27

**Authors:** Sanne P. C. van Oosterhout, Anneke G. van der Niet, Wilson F. Abdo, Marianne Boenink, Jelle L. P. van Gurp, Gert Olthuis

**Affiliations:** 1 Ethics of Healthcare Group, Department of IQ Health, Radboud University Medical Center, PO Box 9101, 6500 HB Nijmegen, The Netherlands; 2 Department of Intensive Care Medicine, Radboud University Medical Center, PO Box 9101, 6500 HB Nijmegen, The Netherlands

**Keywords:** Intensive care unit, Organ donation, Communication, Qualitative research, Clinical ethics

## Abstract

**Background:**

Listening and responding to family concerns in organ and tissue donation is generally considered important, but has never been researched in real time. We aimed to explore in real time, (a) which family concerns emerge in the donation process, (b) how these concerns manifest during and after the donor conversation, and (c) how clinicians respond to the concerns during the donor conversation.

**Methods:**

A qualitative embedded multiple-case study in eight Dutch hospitals was conducted. Thematic analysis was performed based on audio recordings and direct observations of 29 donor conversations and interviews with the family members involved (n = 24).

**Results:**

Concerns clustered around six topics: 1) Life-event of a relative’s death, 2) Dying well, 3) Tensions and fears about donation, 4) Experiences of time, 5) Procedural clarity, and 6) Involving (non-)present family. Most concerns occurred in topics 1 and 2. Clinicians mostly responded to concerns by providing information or immediate solutions, while sometimes acknowledgement sufficed. When concerns were highly charged with emotion, the clinicians’ responses were less frequently attuned to families’ needs. Cues of less clearly articulated concerns gained less follow-up. Then, concerns often remained or reappeared.

**Conclusion:**

The identified concerns and the distinction between clearly and less clearly articulated concerns may prove valuable for clinicians to improve family support. We advise clinicians to engage with a curious, probing attitude to enhance the dialogue around concerns, elaborate on less clearly articulated concerns and identify the informational needs of the family.

**Supplementary Information:**

The online version contains supplementary material available at 10.1186/s13054-024-05198-2.

## Background

Family-centred care in the intensive care unit (ICU) has become important in recent decades [1–4]. Supporting family members is particularly key in the context of potential organ and/or tissue donation, as they are the centre of communication and decision-making [2, 4]. Dutch donation laws and regulations state that clinicians should prevent psychological harms to patients’ families and assess such potential harms based on their professional expertise [5–8]. Emphasis has been placed on providing time and space for families to process both the prognosis and information related to donation. Knowing which specific family concerns arise during donor conversations aids physicians in such cases [5].

Several studies identified, in retrospect, the concerns families experience with donation, emphasising the importance of listening to and addressing these concerns [9–11].Donor conversations occur under acute circumstances that evoke strong emotions and during which families are overwhelmed and cognitively and emotionally unprepared [11–13]. They often struggle to grasp the unexpected impending death of their loved one, and have difficulties to comprehend and respond to all the information [10, 11, 13].Additionally, family members report fears about the donation procedures, or conflicts can come about due to uncertainties about the patients’ donation wishes or pressure surrounding decision-making [10, 11]. Concerns also appear when families interact with healthcare professionals [9]. Families might experience a lack of appropriate, respectful care for their loved one and support for their own needs [10, 11, 13]. Post-donation, families expressed dissatisfaction with aspects such as waiting times or the donation decision [10, 13].

Previous research on families’ concerns in the donation process is primarily focused on concerns in retrospect, often with a focus on decision-making [2, 10, 11, 14, 15]. Additional research is needed to provide insight into how concerns emerge and develop over time, and how professionals’ behaviour affects these concerns. This requires analysis of conversations in real time. Our study aimed to understand how family concerns manifest over time *during and after* the donor conversations. We show *if* and *how* family concerns are addressed in the donor conversations and how clinicians respond to these concerns. We aim to (a) identify family concerns that emerge in the donation process, (b) explore the manifestation of these concerns during and after the donor conversation, and (c) investigate how clinicians respond to the concerns during the donor conversation.

## Methods

### Design and setting

**Fig. 1:**
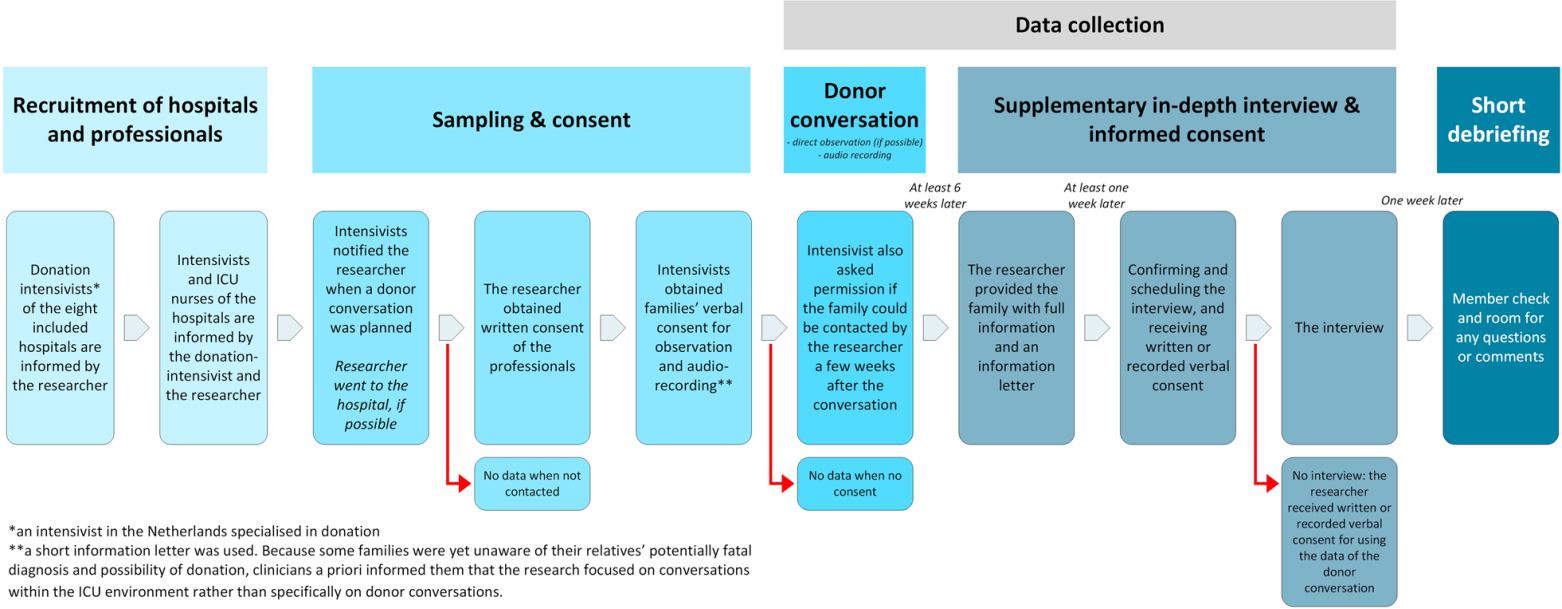
Flow chart with design of the study

The present study is part of a qualitative embedded multiple-case study researching donor conversations in the Netherlands [8]. Cases (n = 29) included audio-recordings, and direct observations of donor conversations when possible, and supplementary in-depth interviews with the family members [16–19]. Figure 1 provides an overview of the design of the study. Data were derived from ICUs of eight Dutch hospitals, including two teaching hospitals and six tertiary university medical centres. Hospitals were selected based on their geographic distribution and annual initiated organ donation rates [20]. 1220 donor conversations took place in the entire country during the inclusion period of our study (February 2021-December 2022), which led to 503 donation procedures.

### Sampling and recruitment

Initially, convenience sampling was utilised due to the unpredictable character of donor conversations. Cases were included in which donation after brain death and/or after circulatory death was discussed. Cases in which only tissue donation was discussed were excluded. Following a comprehensive analysis of six cases, the first author consulted with her supervising committee to discuss criteria for purposive sampling. Aiming for maximum case variation, the sampling strategy was based on case descriptions, donor registration status, and hospital settings [19, 21].

### Data collection

Data were collected during the transition from a voluntary opt-in donor registration system to an active donor registration, opt-out system (Table 1). Cases were included from February 2021 to December 2022, comprising 8 cases within the opt-in registration system and 21 cases within the opt-out registration system leading to a total of 15 successful organ donation procedures. Additional file 1 lists their characteristics. Observations primarily focused on nonverbal cues, and informed the researcher’ preparation of subsequent interviews. Audio-recorders were provided in case the researcher could not be present. All conversations were transcribed verbatim, and field notes were made after every interview and observed case [22].

**Table 1:** Dutch organ donation registry system

Between 1998 and July 2020, the Netherlands operated under an opt-in organ donation registry system. Dutch citizens could register their organ donation preferences and modify them at any time, either digitally or by paper. The available options were: Yes, No, or the family/specific person decides	
If no choice was registered, the law required the family to make the decision	
In July 2020, the system changed to an opt-out model. Citizens can still register the same preferences as before; however, if no choice is made, "presumed consent" is registered, which is considered as a consent for donation. While the family is informed about the registration and method of consent (Yes or presumed consent), they are no longer formally required to provide consent	

Family interviews, conducted at least six weeks after the conversation, explored personal experiences and perspectives using an interview guide (Additional file 2). Twenty-four family members involved in 19 cases consented to an interview (median: 61 min; 36–82 min). Family members from 10 cases did not consent to be interviewed. Interview options included face-to-face, telephone or video interviews, preferably with the closest relative present during the interview. Additional support from other family members was allowed. A short debriefing was conducted via telephone one week after the interview. All interviews were audio recorded and transcribed verbatim. The transcripts were offered for member checking, and interview summaries were provided and approved by all family members [19]. Demographic and clinical data were collected using Castor EDC [23].

### Data analysis

We conducted a thematic analysis within and across cases (donor conversations and interviews with family members) using constant comparison, aiming for general theoretical insights on discussing family concerns [17, 21]. Concerns were conceptualised following the criteria in Additional file 3. The analysis consisted of four steps (Additional file 3) and was facilitated by computer-assisted qualitative data analysis software ATLAS.ti. All recordings were included in the analysis. Standard descriptive statistics were used through IBM SPSS Statistics (version 27). The Consolidated Criteria for Reporting Qualitative Research were used (Additional file 4) [24].

**Fig. 2:**
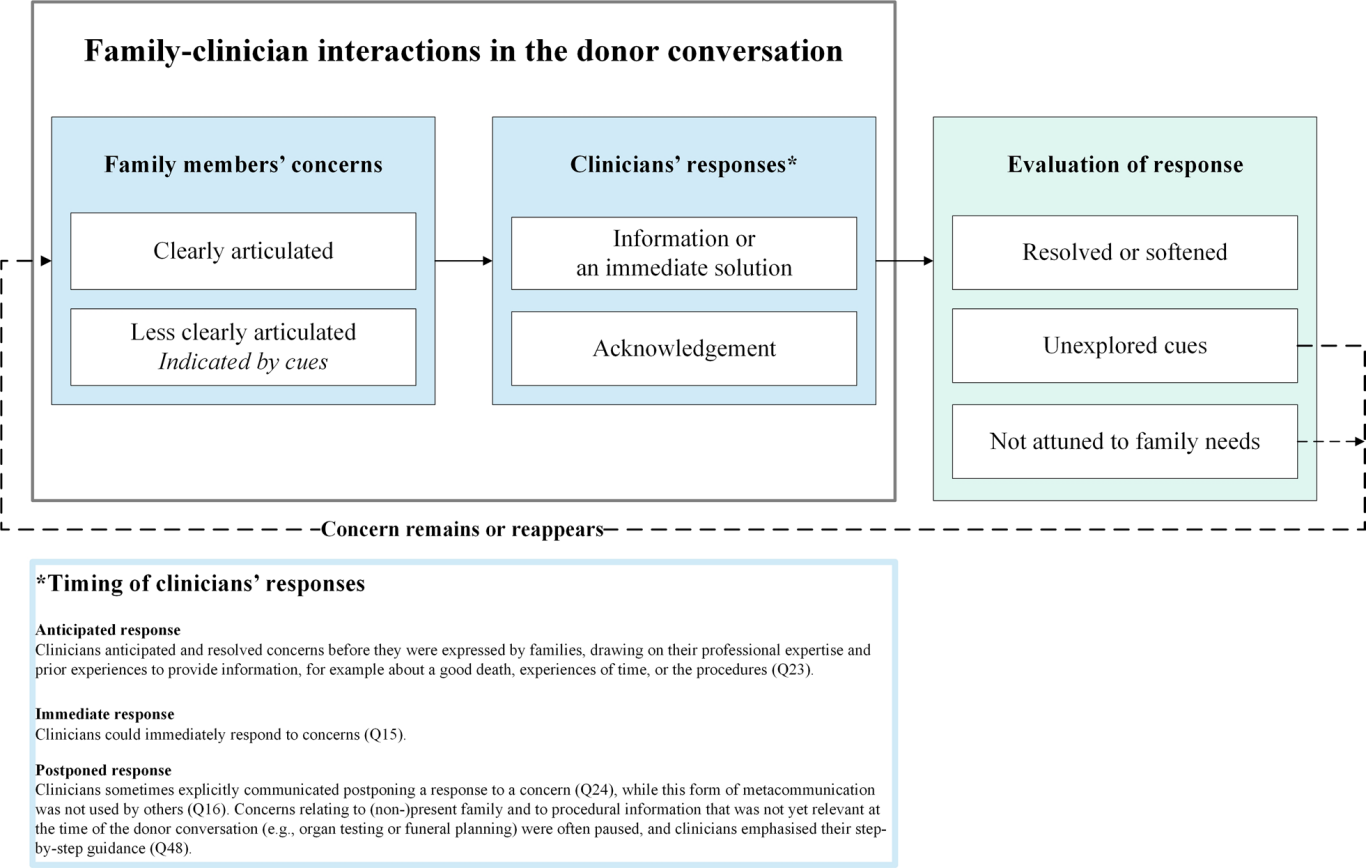
Clinician-family interactions about concerns in the donor conversation

The clinicians’ responses were evaluated based on a combination of the responses of family members in the supplementary interview and our own analysis taking into account Dutch donation laws and regulations (Fig. 2). We based our evaluations on the following assumptions: We assume that family concerns should be acknowledged (as good as possible) and that, when possible, resolving or softening a concern is desirable in order to promote a careful donor conversation. According to Dutch donation laws and regulations, caring for the family members, attuning to their needs, engaging in dialogue with family members, promoting family satisfaction with the donation decision and providing them time and space to process both the infaust prognosis and donation are important moral norms for donor conversations [5–8]. Treating families in a correct manner and with a proper approach where empathy, clear communication and genuine interest are important [5]. Attuning to the family was necessary to evaluate clinician’s response as positive. For example, to verify whether adequate information was provided (as valued in informed consent standards), alignment with the family is necessary.

## Results

Family concerns emerged from the moment the patient was admitted to the hospital, further manifested in the bad news and donor conversations, occurred during the waiting period till death and donation, and can be found afterwards in the supplementary interview. We gathered data during the donor conversation (sometimes including the bad news conversation) and the supplementary interview, the latter also informing us about how the concerns manifested throughout the entire donation process. The most prominent concerns were about the life-event of a relative’s death followed by concerns about dying well (Table 2, also Additional file 5). In Table 2 and throughout the Results section we frequently refer to quotations from the data (transcript of donor conversations and follow-up interviews) to illustrate our observations and analysis (Q1, Q2, etc.). The quotations are provided in Additional file 6.

**Table 2:** Overview of concerns clustered in topics

Topic 1: Life-event of a relative’s death The life-event of a relative’s death can be characterized as a wave of emotions throughout time (Q13). Hope and fear intertwined from hospital admission onwards, and uncertainties and ignorance about the prognosis caused concerns early in the process (Q35). The irreversibility of their loved one’s death, especially sudden and/or unexpected deaths, resulted in shock or lingering disbelief about the relative’s death (Q27). Mostly at the start of the donor conversation, family members expressed concerns about their experienced responsibility for stopping the treatment of their loved one (Q36) and for making a good donation decision, following the wishes of their relative (Q37). The burden of deciding also emerged (Q38), as well as worries about their own physical and mental well-being (Q39).	
Topic 2: Dying well Relatives emphasised the importance of dying well, such as assisting their dying relative (Q11, last part) and be present at the time of death (Q14). Avoiding suffering (Q40), not leaving their loved one alone, and treating their body respectfully and with dignity (Q41).	
Topic 3: Tensions and fears about donation In addition, tensions and fears about donation were expressed during the donor conversation, such as donation “does not feel right (yet)” (Q42) or the fear that the process of donation could not be reversed once agreed upon (Q43).	
Topic 4: Experiences of time Family members also voiced concerns regarding their experiences of time, such as waiting times (Q10/26) or the need for a faster pace in the donation procedure after the donor conversation (Q9/Q10/Q31/Q32).	
Topic 5: Procedural clarity Clarity on procedures, their relative’s wishes, timing surrounding death and donation, and information about the recipient was sought too (Q44).	
Topic 6: Involving (non-)present family Finally, often in the context of pandemic restrictions, families expressed the need for other family members to be present and asked about the opportunities for them to say goodbye to their loved one (Q45).	
Post-donation concerns In addition to the concerns emerging in the donor conversation, families also explicitly voiced post-donation concerns that were experienced when some time had already passed after their relative’s death (**Figure 1** **supplementary interview**). There were families where doubts arose about stopping their relatives’ treatment (Q5). Family members also still had questions (or new questions arose) about the donation and their relative’s death (Q46). Other concerns included regrets, questions about their individual roles in the donation and dying process and about practicalities surrounding their relative’s death (Q47), disappointment with donation outcomes, and doubts about their personal donor registrations .	

Concerns clustered around six topics. Families experienced overwhelming emotions due to the life-event of their relative’s impeding death (topic 1), which hindered them in participating in the donor conversations. They tried to turn off their emotions (Q1) but sometimes felt unable to ask questions or fully process information about donation and death (Q2). Their primary focus was the impeding loss and taking the time to say goodbye without being disturbed by the donation process (Q3) (topics 1 and 2). Additionally, worries existed that stopping active treatment was influenced by the patient’s choice to donate (Q4) or that donation would be initiated against the wishes of their loved one (Q5) (topic 3). Moreover, family members needed time to process the bad news (topic 4), while feeling the pressure to decide about donation and pursue with the conversations and procedures (Q6/7). They expressed being unprepared for and ignorant about (part of) the donation procedures (Q8/Q9) and voiced their need for control over the situation and donation (topic 5) (Q10). Families were also preoccupied with thoughts about (non-)present family members (topic 6). They wanted consensus and alignment with other family members about donation procedures and practicalities surrounding death (Q11), and cared for and protected other relatives (Q12).

Some concerns (e.g., Q13/Q14/Q15) were clearly voiced by families, leaving no room for alternative interpretations. Other concerns were mere hints during the donor conversation. These more tacit concerns were indicated by the family through subtle verbal cues in the donor conversation (Q16/Q17) or in the form of immediate needs (e.g., wanting to go outside, restroom breaks) without fully explaining the underlying concern (Q18). Some concerns were only mentioned during the supplementary interview post-donation and were therefore not mentioned in the previous donor conversation(s) (Q19/Q20).

### Clinician-family interactions about concerns

Families were mostly positive about the support they received from clinicians including clinicians’ attitudes (Q21). Without explicitly asking about ‘concerns’ in the donor conversation, clinicians sometimes inquired what was on family’s minds, mostly during the donation decision-making (e.g., first sentence of Q17). For instance, by asking in general terms whether the family had additional questions (Q22) or needed clarifications. We identified three alternatives of clinician responses to family concerns in the conversations. They provided an anticipated (Q23), an immediate (Q15) or a postponed (Q16/Q24) response (Fig. 2). Figure 2 presents the observed clinician-family interactions regarding concerns and what potentially caused concerns to remain or reappear (Fig. 2*dotted arrow*). Although ICU nurses were mostly observers in the donor conversations, they occasionally provided support to families, for example when clinicians did not (adequately) respond to a concern (Q25).

### Dealing with family members’ concerns

Some concerns of family members arose momentarily and only once (e.g., Q15), while other concerns reappeared multiple times during the donor conversation and afterwards, indicating a sense of urgency or a suboptimal response of clinicians (Q26). The more emotionally loaded the concern was (e.g., life-event), the more likely the concern reappeared anyway during the donor conversation (Q13/Q27). A concern could also be displaced by another that was more prominent at that moment, but reappear again later (or not) (Q28).

To potentially resolve or soften a concern, first the concern needed to be clear, and clinicians’ responses then had to align with families’ needs. We observed that while clinicians frequently provided families opportunity to speak, providing information or immediate solutions were the most common responses to family concerns (Q29). Although some concerns could not be solved, such as those related to the loss of families’ loved one or inextricably linked to the donation (e.g., the waiting period), these concerns could be softened when explored more elaborately, thus enabling careful attuning to family needs. When two concerns were expressed simultaneously, clinicians usually responded to the concern related to practical matters (Q17). While providing information resolved some practical concerns (Q9/Q15), the information was sometimes not aligned with what the family needed (Q30) or with the pace the family preferred (Q9/Q10/Q31/Q32). Sometimes, families sought acknowledgement of their concerns (Q26/Q33) rather than additional information (Q2/Q32) (Fig. 2*not attuned to family needs)*.

While some concerns were clearly articulated by family members, leaving no room for alternative interpretations, other concerns were less clearly articulated. These were only hinted at in the donor conversations and indicated through cues i.e., through more subtle means or in the form of immediate needs, such as wanting to go outside or restroom breaks. Full explanation of the underlying concern was absent and revealed only in the supplementary interview after extensive probing i.e., asking follow-up questions by the researcher. Important to note is that sometimes family members may have given cues noticed by neither the clinician nor the researcher. They also may have experienced concerns that were neither made explicit, nor hinted at by cues. These concerns could not be observed by the clinician nor the researcher.

We observed that clinicians usually did not explore concerns, especially those involving (non-)present family and tensions and fears about donation (Q16/Q17/Q18/Q19/Q20)(Fig. 2*unexplored cues)*. The expressed cues were sometimes misunderstood for concerns related to dying well that could be answered with practical information (Q34). In general, close reading of the conversation transcripts and the supplementary interviews showed that several cues were disregarded by clinicians. While some space was provided for families to voice their concerns more elaborately, families were sometimes also not able to fully express their concerns during the donor conversation (Q19, last part/Q20).

## Discussion

This study is the first to explore how family concerns emerge in real time during the donation process and the donor conversation, and how clinician-family interactions about these concerns take place. It identifies six general clusters of family concerns, inferred from particular concerns voiced in donor conversations and interviews. Some concerns were voiced clearly, others required more explorative questions in order to better understand them. Clinicians were generally inclined to provide information in response to concerns, while acknowledging the concern would sometimes suffice. When cues of more tacitly voiced concerns remained unexplored, a concern was highly charged with emotions, or when clinicians did not attune to families’ needs, concerns often remained or reappeared.

This study adds new insights into family experiences during donor conversations and underlines that they often experience difficulties in voicing their concerns elaborately in real time [15, 25]. Clinicians might not always detect how challenging it can be for family members to express their deepest, most troubling concerns, such as “I don’t want you cutting in my loved one”. Whether all concerns are fully explored seems to be dependent on clinicians’ ability to notice subtle cues, as well as on families’ willingness and ability to express their concerns. Importantly, the unique nature of family concerns is informed by individual experiences with, norms, values, and beliefs related to donation and death. Family concerns are personal but are also embedded in all kinds of societal views on (a good) life and death. The reluctance that is expressed in the concern ‘I don’t want you cutting in my loved one’ shows for example that the human body is not a neutral mechanism but is morally relevant in several ways. This normative relevance is closely connected to the sociocultural context and views on dignity, respect, self-determination, solidarity with others, and dying well [26, 27].

Clinicians should therefore carefully attune to families’ responses and be sensitive to their verbal and nonverbal cues. These skills may be enhanced by experience, but clinicians should also be specifically trained in identifying and addressing these cues. Asking the family open-ended questions about their concerns may invite family members to elaborate on their concerns and creates the opportunity to acknowledge and, where necessary and possible, to address their concerns (Table 3*Example questions)*. This is not specific for donor conversations. A similar conclusion was drawn by Kentish-Barnes and colleagues when discussing concerns of critically ill patients at the ICU [28]. Instead of raising questions that refer to information provision, “concerns” might be addressed more often by clinicians.

**Table 3:** Recommendations for clinicians

Figure 2 presents a schematic account of family-clinician interactions in donor conversations. As research team we have elaborately discussed the implications of the way clinicians noticed and responded to concerns and formulated recommendations to help improve interaction during donor conversations. We have discussed these recommendations at several occasions with stakeholders. ***Reappearing of concerns*** • Be aware that some highly emotionally charged or urgent concerns might reappear anyway. This also applies for concerns that are displaced by other concerns. ***Clinician’s responses*** • Active listening is crucial. • Verify whether the concern *can* potentially be solved or softened with information or if acknowledgement is more appropriate. Note that not all concerns can be solved. • Note that providing information is mostly sufficient when concerns are based on missing or misinterpreting information, for example about the donation procedures and impending death. Providing acknowledgement is mostly sufficient when concerns are rather based on the irreversibility, suddenness and time pressure of the situation. Also note that providing information can be combined with providing acknowledgement. • If information is needed, check *what* information the family needs, and whether that information is adequate and provided in the right pace. ***Exploration of cues*** • Pick up potential cues of less clearly articulated concerns and ask questions** to elaborate and explore these concerns. • When asking about concerns, be aware of questions such as “what are your questions?” or “do you need clarification?” as these refer to information provision. Furthermore, language is key here: asking about family opinions (on organ donation) is not advised, as this might lead to misunderstandings about families’ role in donation decision-making, i.e. families might feel the decision to donate is theirs to make irrespective of the donor registration of the patient. • Be aware of: - your timing: the donor registration is leading and should be communicated and established with the family prior to your own exploration of family concerns. - your own assumptions about concerns and whether you assume the concern to be already clearly articulated. - the need to verify whether your assumptions are in line with the concerns the family members have. - the possibility that some family members are not (yet) able to fully express their concerns, despite your explorative questions. **Example questions:** - What are your concerns? - What is your main concern at this moment? - Where are your thoughts going now? - What do you mean with “X”? - What exactly is your fear regarding “X”?	

Dialogue about concerns is complicated by the asymmetry inherent in interactions between healthcare professionals and families. Families are dependent on clinicians’ expertise about donation and their guidance in the process and often lack the interpretative resources of healthcare professionals [29, 30]. Clinicians serve as information conduits for donation decision-making and are trained with informed consent standards as central principle in their professional conduct [31, 32]. Meanwhile, the topic of donation is increasingly regulated by legal frameworks that also mandate explanation [5, 6]. Consequently, clinicians may be inclined to primarily respond to concerns with information. However, it is crucial to note that families comprehend only half of the information given to them at the intensive care, [33]. and even if they understand it simply offering information may not always address or ease the concerns. The donation conversation may be further complicated by the strong focus on medical issues in clinical practice, discouraging interest in personal experiences and stories, despite the friendliness and courteousness that are often present [34].

We emphasise that establishing a kind of “common ground”, i.e., a mutual understanding about what the family is concerned about, is important for a follow-up dialogue about concerns [35]. As our study highlights, a clinical encounter such as a donor conversation is an inter-relational (and thus: moral) encounter between family members and intensive care staff [36]. To find “common ground” in this conversation the follow-up dialogue requires an exploration of a family’s life world, the taken-for-granted norms, values, knowledge and expectations that shape their understanding of donation. Three relevant, intertwined dimensions of their lifeworld should be taken into account in this exploration [37]: 1/ the objective dimension focussing on knowledge (i.e., the medical situation, facts about donation procedure, donor registration), 2/ the social dimension in which relations are central (i.e., mutual family relations, values that guide social interaction of donor and family), and 3/ the subjective dimension which includes the worldview of those involved (i.e., the intentions, thoughts, emotions and potential donor’s wishes).

A curious, probing approach based on these three interconnected dimension to establish common ground might prevent inflicting psychological harms on families, which is a an important norm in Dutch donation laws and regulations. Earlier research showed that in conversations where families become highly emotional, uttering an empathic reaction should be supplemented with dedicated time to explore what families are going through and how they feel [38]. We endorse this approach, especially considering the emotionally charged, personal and sometimes less clearly articulated character of concerns, as revealed in the present study. Taking an inquisitive approach to concerns in the donor conversation, similar to the approach an interviewer adopts in in-depth interviews, [39] can make concerns more clear and increase the likelihood of resolution or mitigation. It may also reduce the risk of potential conflicts, [38] and potentially enhance family satisfaction and donation consent rates [15].

Once donor wishes are established clinicians should verify *whether* and *what* specific information is needed, as well as carefully listen to what families express (Table 3). Furthermore, engaging in metacommunication to understand family needs and counteract clinicians’ own inclinations might also be beneficial, although time constraints may pose practical limitations. Since it may be challenging for clinicians to combine the role of information provider valuing informed consent with the curious, probing attitude to establish common ground, this should be incorporated in training.

Our study provides unique insights into how families’ concerns emerge during the organ donation process and into the clinician-family interactions about these concerns. Using real-time donor conversations with supplementary interviews is the main strength of our study. Although the study was conducted in a specific Dutch legal setting our findings can also be of value to countries with other legal systems. The six identified clusters of concerns were found in both the opt-in and opt-out cases. This suggests that this article’s concerns are not related to a specific legal registration system. The study has several limitations. Despite our efforts to translate information letters, we did not succeed in including cases with families from minority groups and various cultural backgrounds, for whom concerns might be different. Since post mortal donation can be a sensitive topic, there is a possibility of social desirability bias. We also potentially missed some concerns and consecutive responses, as we could not include non-verbal communication in our analysis when no direct observation took place, although this minimized the intrusiveness of the data collection. Clinicians’ responses might also be underreported, as we analysed responses in reaction to identified concerns during the donor conversations. They might have addressed concerns outside the donor conversation, in (in) formal (bedside) conversations with families that we did not register. Furthermore, although ICU nurses only briefly participated in the donor conversations, they might also have responded to the concerns during informal bedside conversations. A suggestion for future research is to shadow families more closely to investigate how their concerns develop during the donation process, which also provides the opportunity to ask clinicians directly about their response to identified concerns for additional verification.

## Conclusion

Our study shows that family concerns manifest throughout the donation process. These concerns are highly personal and require clinicians to carefully attune to them in donor conversations. Clinicians’ awareness of family concerns, including those communicated through cues in the donor conversations, is key. The clusters of family concerns we identified can provide guidance for clinicians in future communication with families. Moreover, our analysis of the process of clinician-family interactions shows that while these may result in resolution and mitigation of concerns, concerns may also remain or reappear, especially when cues remain unexplored or clinicians’ responses are not attuned to family needs. We strongly recommend clinicians to search for and explore less clearly articulated concerns. This requires being sensitive for cues and adopting a probing approach, asking follow-up questions rather than providing information and asking informed consent (Table 3). Active listening and acknowledging family concerns are crucial.

## Supplementary Information

Additional file1 (PDF 210 kb)

Additional file2 (PDF 111 kb)

Additional file3 (PDF 78 kb)

Additional file4 (PDF 128 kb)

Additional file5 (PDF 160 kb)

Additional file6 (PDF 338 kb)

## Data Availability

The data supporting the findings of the current study are available from the corresponding author on reasonable request.

## Abbreviation

ICU: Intensive Care Unit
