# Supplementary material for: Family concerns in organ donor conversations: a qualitative embedded multiple-case study
Source: Crit Care. 2024 Dec 27;28:434. doi: 10.1186/s13054-024-05198-2 (PMC11673370; doi:10.1186/s13054-024-05198-2)
Supplement: Supplementary file 1 — Additional file1 (PDF 210 kb) [file 13054_2024_5198_MOESM1_ESM.pdf]

Manuscript title:

Family concerns in organ donor conversations: a qualitative embedded multiple-case study

Corresponding author: Gert Olthuis. Gert.olthuis@radboudumc.nl

SUPPLEMENTARY INFORMATION

Additional file 1a. Characteristics of cases of organ and tissue donor conversations (n=29), patients (n=29) and family members (n=24).

Characteristic	Number (%)	Median (range)
**Family donor conversations (n=29)**		
**Moment during the day**		
Morning	5 (17)	-
Afternoon	14 (48)	
Evening	6 (21)	
At night	4 (14)	
**Decoupled, yes**[a]	20 (69)	-
**Duration donor conversation(s) (minutes in total)**[b]	-	20 (5-55)
**Number of attendees**		
Healthcare professionals and researcher	-	3 (2-5)
Family members and friends of the patient		3 (1-7)
**Type of donation – discussed**[c]		-
Donation after Circulatory Death (DCD)	23 (79)	
Donation after Brain Death (DBD)	20 (69)	
Tissue donation	15 (52)	
**Patients (n=29)**		
**Gender, female**	11 (38)	-
**Age (years)**	-	61 (18-72)
**Critical injury**		-
Aneurism	1 (3)	
Cardiac Arrest	1 (3)	
Cerebral Vascular Accident	2 (7)	
Intracranial Haemorrhage[d]	13 (45)	
Myocardial Infarction	2 (7)	
Not known (Pulmonary Embolism)	1 (3)	

Traumatic Brain Injury[e]	8 (26)	
Thrombosis with Thrombocytopenia Syndrome	1 (3)	
**Length of stay (days)**	-	2 (0.1-23)
**Donor registration**		
Consent	8 (28)	-
Presumed consent	8 (28)	
Family consent	6 (21)	
Unregistered	7 (24)	
**Donation - result**		
Initiated and successful procedure[f]	15 (52)	-
DCD	8	
DBD	7	
Initiated procedure, but no successful procedure[g]	7 (24)	
No initiated procedure due to family opposition	7 (24)	
**Family members (n=24)**		
**Gender, female**	16 (67)	-
**Age (years)**	-	55 (23-74)
**Educational level**		-
Primary education	3 (13)	
Secondary education	10 (42)	
Higher education	11 (46)	
**Relationship with the deceased patient**		-
Adult child	4 (17)	
Daughter	2	
Son	2	
Ex-spouse/partner	1 (4)	
Parent	7 (29)	
Father	3	
Mother	3	
Mother-in-law	1	
Sibling	5 (21)	
Sister	3	
Brother	1	
Brother-in-law	1	
Spouse/partner	7 (29)	
**Personal donor registration**		-
No consent	2 (8)	
Consent	11 (46)	
Presumed consent	2 (8)	
Family consent	6 (25)	

Unregistered	3 (13)	
**Days since patient's death**	-	66 (39-152)
**Type of interview**		-
Face-to-face	6 (25)	
At home	2	
Work environment of family member	1	
Work environment of researche	3	
Telephone	7 (29)	
Video	11 (46)	
**Duration interview (minutes)**	-	61 (36-82)

[a]From the bad news conversation.

[b]Rounded to minutes. When the donor conversation is not decoupled from the bad news conversation, the duration includes the duration of the bad news conversation and the donor conversation together. The donor conversation of case 8 and 10 was separated over two conversations.

[c]Various types of donations could be discussed in the conversations.

[d]One case also with a secondary cardiac arrest.

[e]With or without secondary Anoxic/Hypoxic Brain Injury.

[f]Tissues were also donated in four cases (two DBD cases, one DCD case and one case without organ donation)

[g]Initiated procedure, but no donation because organs and, if applicable, tissues were not usable/rejected, or the patient did not die within two hours (for DCD)

Additional file 1b. More detailed characteristics of cases of family donor conversations, patients and family members.

Case (ID)	Family donor conversations (n=29)				Patients (n=29)				Family members (consenting interview, n=24)	
	**Moment during the day[a]**	**Decoupled[b]**	**Duration (minutes)[c]**	**Other attendees**	**Sex/age**	**Critical injury**	**Length of stay (days)**	**Type of donation discussed (result)**	**Relation to (potential) donor*** **	**Sex/age**
**Consent cases**										
**14**	D	Yes	20	Donation intensivist, ICU nurse, two daughters, son, two sons-in-law	F 57	ICH	7	DBD, DCD, tissue (DCD)	-	-
**16**	B	Yes	16	Donation intensivist, ICU nurse, friend	M 63	Not known (PE)	1	DBD, DCD, tissue (DBD, tissue)	Spouse/partner	F 66
**21**	C	Yes	19	Fellow, ICU nurse, medical intern, son, friend	F 60	ICH	4	DCD, tissue (DCD, tissue)	-	-
**26**	A	Yes	22	Intensivist, ICU nurse in training, medical intern, sister, brother-in-law	M 60	MI	14	DBD, DCD (DCD)	Spouse/partner	F 72
**27**	B	No	19[d]	Intensivist, resident, ICU nurse, two daughters, two sons,	M 64	ICH	1	DBD, DCD, tissue (DCD)	Spouse/partner	F 61

				brother-in-law						
**28**	D	Yes	30	Fellow, resident not in training, ICU nurse, spouse/partner, two sons	M 71	ICH & CA	0.1	DBD, DCD (no donation[e])	-	-
**29***	A	No	32	Fellow, ICU nurse, psychiatrist	F 25	CA	2	DBD, DCD, tissue (DBD)	Mother<br>Father	F 53<br>M 60
**3†***	B	Yes	22	Fellow, ICU nurse, ICU resident not in training, spouse/partner, son and daughter of spouse/partner	M 62	TBI	6	DBD, DCD (no donation[e])	-	-
**Presumed consent cases**										
**4***	D	Yes	1: 15<br>2: 14	Donation intensivist, ICU nurse, two sons, daughter-in-law, sister-in-law	M 62	TBI, HBI	1	DBD, DCD (no donation[f])	-	-
**8***	B	No	39	Intensivist, ICU nurse, neurosurgeon, daughter	M 72	TBI	1	DCD, tissue (no donation[e])	Spouse/partner	F 72
**11***	A	No	1: 35<br>2: 21	Intensivist, ICU nurse in training, fellow, spouse/partner, son, daughter	M 68	ICH	0.5	DBD, DCD (DCD)	-	-

**15**	B	No	19	Fellow, ICU nurse, daughter's boyfriend, mother	F 60	ICH	1	DBD (DBD)	Daughter	F 23
**18**	C	Yes	12	Intensivist, ICU nurse, ICU nurse in training, spouse/partner, son, daughter, daughter-in-law, son-in-law, brother, sister-in-law	M 53	ICH	0.3	DBD, tissue (no donation[e])	-	-
**19**	B	Yes	10	Intensivist, fellow, ICU nurse, ICU nurse in training, ODC, mother	F 41	CVA	1.5	** (no donation[f])	Spouse/partner<br>Mother-in-law	M 40<br>F 64
**20**	B	Yes	14	Fellow, ICU resident not in training, ICU nurse, two sisters, daughter, niece	M 61	CVA	2	DBD, DCD, tissue (DCD)	-	-
**23**	B	Yes	18	Donation intensivist, ICU nurse, medical intern, spouse/partner, daughter, son-in-law	M 63	TBI	4	DCD (DCD)	-	-
**Family consent**										
**10***	D	Yes	32	Intensivist, ICU nurse in training,	M 61	Aneurism	1	DBD, DCD, tissue	Daughter	F 30

				spouse/partner, son, daughter				(no donation[f])		
**13***	C	No	1: 18<br>2: 11<br>3: 13	Donation intensivist, intensivist, ICU nurse in training	F 19	TBI & HBI	3	DBD, DCD, tissue (DBD)	Mother<br>Father	F 49<br>M 51
**17**	B	Yes	13	Intensivist, fellow, ICU nurse, spouse/partner, father, mother, brother-in-law, son (<18y)	F 47	ICH	2	** (no donation[f])	Sister	F 43
**22**	B	Yes	5	Physician assistant, ICU nurse, intensivist, spouse/partner, daughter, son-in-law	M 60	ICH	5	** (no donation[f])	-	-
**25**	A	Yes	18	Intensivist, ICU nurse, stepson, niece, friend	F 61	ICH	12	DBD, DCD, tissue (DBD, tissue)	Sister	F 74
**9**[†*]	A	No	26	Intensivist, fellow, ICU nurse, neurosurgeon, nephew with his partner	M 64	ICH	1	DBD, DCD, tissue (DBD)	Spouse/partner	F 62
**Unregistered**										
**24**[g]	B	Yes	20	Fellow, ICU nurse, ODC	F 18	TBI	0.5	DBD, DCD (DBD)	Mother<br>Father	F 45<br>M 52
**1**[†*]	C	Yes	28	Fellow, ICU nurse, two	M 60	TBI	23	DBD, DCD, tissue	Spouse/partner	F 57

				sisters-in-law, niece				(no donation[f])		
**2**[†]	B	Yes	16	Fellow, ICU nurse, ICU resident not in training, one sister, two brothers	F 60	ICH	8	DBD, DCD (no donation[e])	Sister<br>Brother-in-law[h]	F 62<br>M 70
**5**[†*]	C, D	No	35	Donation intensivist, ICU nurse, spouse/partner	F 62	ICH	11	DBD, DCD, tissue (DCD)	Son	M 33
**6**[†*]	C	Yes	36	Donation intensivist, ICU nurse	M 68	TBI & ABI	3	DCD (no donation[e])	Brother	M 58
**7**[†]	B	Yes	10	Donation intensivist, ICU nurse, three children (one <18y)	M 63	TTS	1	** (no donation[f])	Ex-spouse/partner	F 53
**12**[†]	B	No	21	Intensivist, fellow, resident, ICU nurse, son, daughter-in-law, father	M 55	MI	1	DCD, tissue (tissue; no organ donation[e])	Son	M 26

ABI: Anoxic brain injury, DCD: Death after Circulatory Death, DBD: Donation after Brain Death, CA: Cardiac Arrest, CVA: Cerebral vascular accident, ICH: Intracranial haemorrhage, ICU: Intensive Care Unit, HBI: Hypoxic brain injury, MI: Myocardial Infarction, ODC: organ donor coordinator (ODCs in the Netherlands are generally involved after the donor conversation, but clinicians may request their participation in the conversation based on ODCs' donation expertise), PE: Pulmonary Embolism, TBI: Traumatic Brain Injury, TTS: Thrombosis with thrombocytopenia syndrome

[a]Moment during the day, A: Morning, B: Afternoon, C: Evening, D: At night.

[b]From the bad news conversation.

[c]Rounded to minutes. When the donor conversation is not decoupled from the bad news conversation, the duration includes the duration of the bad news conversation and the donor conversation together. The donor conversation of case 4, 11 and 13 was separated over two conversations.

[d]Not the entire conversation is recorded due to low battery level of the audio-recorder.

[e]Initiated procedure, but no donation because organs and, if applicable, tissues were not usable/rejected, or the patient did not die within two hours (for DCD)

[f]No initiated procedure, because family opposed in the donor conversation (not patient's wish or family's potential psychological harm) or the family decides in case of the donor registration of "family consent".

[g]Due to the fact that the patient had just turned 18 years old and had not yet received (all the) letters of the Dutch government about the amended Donor Act requesting donor registration.

[h]This person (brother-in-law) was not present at the donor conversation. He supported the sister (his wife) in the interview.

*The researcher directly observed and audio-recorded the donor conversation.

**No information about donation, only mentioning the option for organ and tissue donation. In case 22, the option of DCD is suggested, but the family immediately states that the patient did not want to be a donor.

***When multiple family members are listed in one case, they participated in one interview together.

†These cases were included in the previous Dutch opt-in donor system.
