# Supplementary material for: Family concerns in organ donor conversations: a qualitative embedded multiple-case study
Source: Crit Care. 2024 Dec 27;28:434. doi: 10.1186/s13054-024-05198-2 (PMC11673370; doi:10.1186/s13054-024-05198-2)
Supplement: Supplementary file 2 — Additional file2 (PDF 111 kb) [file 13054_2024_5198_MOESM2_ESM.pdf]

Manuscript title:

Family concerns in organ donor conversations: a qualitative embedded multiple-case study

Corresponding author: Gert Olthuis. Gert.olthuis@radboudumc.nl

SUPPLEMENTARY INFORMATION

Additional file 2. Topic list for family interviews.

Ideally, nondirective, open-ended questions were asked. Questions were adjusted with specific case information, as all interviews applied to one specific case. Direct observations and audio-recordings were used to inform the researcher about which elements to emphasise in the interviews.

1) General review of the donor conversation and donation process

This part of the interview informed the researcher about the quality and quantity of memories of the family and established an interviewer-interviewee relationship. It gave space to family's experiences and could already identify major concerns – if any – for the family. Recalling the donor conversation and the timeline of events was especially hard for some family members. The researcher helped with reconstructing events where needed, but mostly probed the experiences that the family did remember.

- Review of experiences about donation in general and the donor conversation(s) in particular

1) Before the donor conversation

This part was mainly used to gain information on the case and participants in the donor conversation.

- Case information
- Prior experience and familiarity with Intensive Care Unit (ICU) clinician(s) and nurses
- Preparation for and expectations of the donor conversation including the acceptance of the infaust prognosis of the potential donor and timing of the donor conversation
- Prior knowledge and experience with donation

2) During the donor conversation

The interview mainly focused on this part.

- Patient's donor registration, donation wishes and its implications for the conversation including family's awareness of this
- Roles of the participants (clinician[s], ICU nurse and family member[s])
- Information provision
- Donation decision and, if applicable, resolving differences and credible oppositions
- Family concerns and needs

The researcher also tried to gain information on how specific concepts in the "Kwaliteitsstandaard Donatie" (Quality Standard for Donation [QSD])(e.g., "dialogue", "consensus", "proper treatment", "professional judgment") were applied.

3) After the donor conversation

- Aftercare regarding donation
- Final reflection and review
- Personal opinion on donation and the amended Donor Act
