# Supplementary material for: Family concerns in organ donor conversations: a qualitative embedded multiple-case study
Source: Crit Care. 2024 Dec 27;28:434. doi: 10.1186/s13054-024-05198-2 (PMC11673370; doi:10.1186/s13054-024-05198-2)
Supplement: Supplementary file 3 — Additional file3 (PDF 78 kb) [file 13054_2024_5198_MOESM3_ESM.pdf]

Conceptualisation of family concerns(10)

1. Concerns indicate a certain trouble or effort and point to a difficulty. Concerns reveal what is not going smoothly and cause feelings of worry.
2. Concerns have a personal character; they occupy a family member at a particular moment and force him or her to personally resolve the problem. Concerns are not about what families in general have to overcome but are more precise, personal, contextual, and situational and are shaped by previous health care experiences of individual family members.
3. Concerns impose themselves with a certain urgency. It is hard for family members not to be caught up in the efforts to deal with a concern. They cannot ignore, omit or neglect concerns.
4. Concerns inevitably require effort. For family members, dealing with concerns is not self-evident; it often takes courage, determination, patience, improvisation, talent, etc, to manage them and to get a grip on the situation.

Several cues identified family concerns, such as explicitly voicing the concerns in the donor conversation, showing concerns through behaviour (e.g., posture, emotional expressions, agitation) or revealing them during the supplementary interview.

Case analysis started with identifying concerns in the family interview followed by those in the corresponding donor conversation(s). Identified concerns in the interview sensitised the researcher to identify related and other concerns in the donor conversation(s).

Step 1. Open coding, family concerns

- Two members of the research team (SO and AN) independently open coded 10* opt-out cases (10 donor conversations triangulated with 7 family interviews), which were critically discussed and carefully compared after every case to resolve disagreements and reach consensus.(1, 2)
- SO open coded another 3 opt-out cases (3 donor conversations), while repeatedly consulting AN to discuss uncertainties.(1)
- Cases were selected based on availability of family interviews and diversity in donor registration, case description and geographical location.
- The constant comparison method was used.(3)
- The identified codes formed the basis for the preliminary coding scheme.

*These cases were not the *first* cases collected.

Step 2. Open coding, clinicians' responses

- SO and AN independently open coded another 4 cases with family interviews (2 opt-out and 2 opt-in). After discussion and agreement on the identified concerns, they independently analysed clinicians' responses to the concerns in these donor conversations.(1, 2)
- SO open coded another 7 cases (7 donor conversations with clinicians' responses [3 opt-out, 4 opt-in] triangulated with 3 family interviews) with the preliminary coding scheme, while repeatedly consulting AN to discuss uncertainties.(1)
- Cases were selected to increase case and geographical variation.
- Uncertainties in procedures and coding were continuously and intensively peer reviewed throughout the process of analysis with four members of the research team (SO, AN, JG, GO) to strengthen the analytical rigor.(1, 4)

The data was collected and analysed iteratively, allowing us to continuously reflect on the data and ensure data saturation.(5) We adjusted the interview guides based on emerging ideas and iteratively developed a coding scheme following steps 1 to 4 (**Additional file 4**).(2, 3, 6-9) As the analysis proceeded, codes were thoroughly removed from, added to, adapted, or combined in the coding scheme. Open codes were integrated into axial codes, categories, and finally into themes.

Step 3. Selective coding

- SO selectively coded the remaining 5 cases (5 donor conversations with clinicians' responses [3 opt-out and 2 opt-in] triangulated with 5 family interviews) to examine whether new codes emerged from the analysis.(2) They continued discussing their disagreements and uncertainties until consensus was reached.(1)
- SO also selectively analysed clinicians' responses of the 13 cases in Step 1 Open coding, family concerns.(2)
- Data collection was concluded at data saturation: when the data stopped producing value-added theoretical information.(5)

Step 4. Theme development

- Themes were developed inductively.(2, 3, 6-9)
- Six members of the research team (SO, AN, FA, JG, GO, MB) elaborately analysed and peer reviewed the coding scheme to identify overarching patterns.(1, 4)
- As member check, themes were critically reviewed in two expert meetings with stakeholders in the field of organ and tissue donation.(1, 4)

References

1. Barbour RS. Checklists for improving rigour in qualitative research: a case of the tail wagging the dog? BMJ. 2001;322(7294):1115-7.
2. Strauss A, Corbin JM. Basics of qualitative research: Grounded theory procedures and techniques. Thousand Oaks, CA, US: Sage Publications, Inc; 1990. 270- p.
3. Glaser BG. The Constant Comparative Method of Qualitative Analysis. Social Problems. 1965;12(4):436-45.
4. Barry CA, Britten N, Barber N, Bradley C, Stevenson F. Using reflexivity to optimize teamwork in qualitative research. Qual Health Res. 1999;9(1):26-44.
5. Morse JM. The significance of saturation. Sage publications Sage CA: Thousand Oaks, CA; 1995. p. 147-9.
6. Miles MB, Huberman AM, Saldaña J. Qualitative data analysis: A methods sourcebook: Sage publications; 2018.
7. Elo S, Kyngäs H. The qualitative content analysis process. J Adv Nurs. 2008;62(1):107-15.
8. Hsieh H-F, Shannon SE. Three Approaches to Qualitative Content Analysis. Qual Health Res. 2005;15(9):1277-88.
9. Malterud K. Qualitative research: standards, challenges, and guidelines. Lancet. 2001;358(9280):483-8.
10. Olthuis G, Prins C, Smits MJ, van de Pas H, Bierens J, Baart A. Matters of concern: a qualitative study of emergency care from the perspective of patients. Ann Emerg Med. 2014;63(3):311-9.e2.
