# Supplementary material for: Family concerns in organ donor conversations: a qualitative embedded multiple-case study
Source: Crit Care. 2024 Dec 27;28:434. doi: 10.1186/s13054-024-05198-2 (PMC11673370; doi:10.1186/s13054-024-05198-2)
Supplement: Supplementary file 4 — Additional file4 (PDF 128 kb) [file 13054_2024_5198_MOESM4_ESM.pdf]

Manuscript title:

Family concerns in organ donor conversations: a qualitative embedded multiple-case study

Corresponding author: Gert Olthuis. Gert.olthuis@radboudumc.nl

SUPPLEMENTARY INFORMATION

Additional file 4. Consolidated criteria for reporting qualitative studies (COREQ): 32-item checklist.

Developed from:

Tong A, Sainsbury P, Craig J. Consolidated criteria for reporting qualitative research (COREQ): a 32-item checklist for interviews and focus groups. Int J for Qual Health Care 2007;19(6):349-57.

**Item number**	**Guide questions/description**	**Reported in section**
**Domain 1: Research team and reflexivity**		
Personal Characteristics		
1. Interviewer/facilitator	Which author/s conducted the interview or focus group?	Contributions on Unblinded title page
2. Credentials	What were the researcher's credentials? E.g., Ph.D., M.D.	Untitled title page
3. Occupation	What was their occupation at the time of the study?	Untitled title page
4. Gender	Was the researcher male or female?	Untitled title page (name)
5. Experience and training	What experience or training did the researcher have?	SO is a qualitative researcher
Relationship with participants		
6. Relationship established	Was a relationship established prior to study commencement?	N/A
7. Participant knowledge of the interviewer	What did the participants know about the researcher? E.g., personal goals, reasons for	Information letter and consent form

	conducting the research	
8. Interviewer characteristics	What characteristics were reported about the interviewer/facilitator? E.g., bias, assumptions, reasons and interests in the research topic	N/A
**Domain 2: Study design**		
Theoretical framework		
9. Methodological orientation and Theory	What methodological orientation was stated to underpin the study? E.g., grounded theory, discourse analysis, ethnography, phenomenology, content analysis	Methods, Data analysis
Participant selection		
10. Sampling	How were participants selected? E.g., purposive, convenience, consecutive, snowball	Methods, Case inclusion.
11. Method of approach	How were participants approached? E.g., face-to-face, telephone, mail, email	WFA and SO called the donation intensivists* of the participating hospitals. These donation intensivists and the researcher informed all clinicians verbally and in writing about the study.
12. Sample size	How many participants were in the study?	Results, first paragraph
13. Nonparticipation	How many people refused to participate or dropped out? Reasons?	Results, first paragraph
Setting		
14. Setting of data collection	Where was the data collected? E.g., home, clinic, workplace	Methods, Design and setting and Data collection
15. Presence of nonparticipants	Was anyone else present besides the participants and researchers?	Results, Table 1
16. Description of sample	What are the important characteristics of the sample? E.g., demographic data, date	Results, first paragraph, Table 1, and Additional file 5
Data collection		
17. Interview guide	Were questions, prompts, guides provided by the authors? Were they pilot tested?	Methods, Data collection, and Additional file 2
18. Repeat interviews	Were repeat interviews carried out? If yes,	N/A

	how many?	
19. Audio/visual recording	Did the research use audio or visual recording to collect the data?	Methods, Data collection
20. Field notes	Were field notes made during and/or after the interview or focus group?	Methods, Data collection, and Additional file 2
21. Duration	What was the duration of the interviews or focus group?	Results, first paragraph, and Additional file 5
22. Data saturation	Was data saturation discussed?	Methods, Data Analysis
23. Transcripts returned	Were transcripts returned to participants for comment and/or correction?	Methods, Data collection
**Domain 3: Analysis and findings**		
Data analysis		
24. Number of data coders	How many data coders coded the data?	Figure 1
25. Description of the coding tree	Did authors provide a description of the coding tree?	Additional file 3
26. Derivation of themes	Were themes identified in advance or derived from the data?	Methods, Data analysis, and Figure 1
27. Software	What software, if applicable, was used to manage the data?	Methods, Data analysis
28. Participant checking	Did participants provide feedback on the findings?	Methods, Data collection, and Figure 1
Reporting		
29. Quotations presented	Were participant quotations presented to illustrate the themes/findings? Was each quotation identified? E.g., participant number	Results, description of the themes, and Additional file 6
30. Consistency of data and findings	Was there consistency between the data presented and the findings?	Results and Additional file 6
31. Clarity of major themes	Were major themes clearly presented in the findings?	Results, description of the themes
32. Clarity of minor themes	Is there a description of diverse cases or discussion of minor themes?	Results, description of the themes

*A donation intensivist is an intensivist with a specific focus on donation.
