# Supplementary material for: Family concerns in organ donor conversations: a qualitative embedded multiple-case study
Source: Crit Care. 2024 Dec 27;28:434. doi: 10.1186/s13054-024-05198-2 (PMC11673370; doi:10.1186/s13054-024-05198-2)
Supplement: Supplementary file 5 — Additional file5 (PDF 160 kb) [file 13054_2024_5198_MOESM5_ESM.pdf]

Manuscript title:

Family concerns in organ donor conversations: a qualitative embedded multiple-case study

Corresponding author: Gert Olthuis, gert.olthuis@radboudumc.nl

SUPPLEMENTARY INFORMATION

Additional file 5. Coding scheme of the topics of family concerns for cases and supplementary interviews with family members.

**Topic of family concern**	**(Sub)Categories[a]**
1) Life-event of a relative's death	**I* live between hope and fear**<br>- I am ignorant and experience uncertainties before the bad news conversation<br>- I still have some hope for recovery: I have worries for when my relative comes home<br>- I am overwhelmed by the bad news and the whole situation (shock, disbelief)<br>- I am overwhelmed my having to make a donation decision: it is a dilemma<br><br>**I am constantly overwhelmed by my emotions**<br>- I am angry about my relative's cause of death<br>- My relative's death leaves me with intense grief<br>- My emotions are overwhelming, I have no room for rational thinking (such as processing information and asking questions)<br>- I try to turn off my emotions as a coping mechanism during the donor conversation(s) and before the donation procedure<br>- I have concerns about my own physical and mental resilience<br><br>**I feel I have a responsibility to bear**<br>- I do not want the responsibility for my relative's death<br>- I experience a responsibility for the donation decision<br><br>**I have doubts about stopping treatment in the first place**<br>- I have personal doubts and questions about stopping treatment in the first place<br>- I have doubts about the skills of the clinicians (also regarding the donation)
2) Dying well	**I do not want my relative to suffer (I do not want to unnecessarily prolong life and further suffering)**<br>- I do not want my relative to suffer<br>- I want the donation procedure to proceed carefully and ethically<br><br>**I want to keep my relative's image intact/undisturbed**<br>- I want my relative to look the same as he/she/X was (reminder of my relative's appearance)<br>- I want to say goodbye without any tubes<br>- I want to remember pleasant moments with my relative

	**I want to assist my dying relative (for my relative and for myself)**<br>- I do not want anyone else touching my relative<br>- I want privacy, space and time when saying goodbye<br>- I want to be there at the moment of death<br>- I would have liked to (or want to) be/sit with my relative for a longer period of time and not leave him/her/X alone
3) Tensions and fears about donation	**Now that I am personally involved in donation, the donation does not feel good (yet)**<br><br>**I fear clinicians' motivations for stopping treatment and pursue donation**<br>- I am afraid of a conflict of interest: that my relative is killed for his/her/X organs<br>- I am afraid that donation is continued despite my relative's objection for donation<br><br>**The experience of a donation procedure has its effects on me and disappoints me**<br>- I want to hear from the donor recipients and had expected more about that<br>- In retrospect, I would have liked more organs and tissues to be donated<br>- Afterwards, I have a strong aversion to donation: I would never do it again.<br>- Now that I have experienced a donation procedure, I have doubts about my own donor status
4) Experiences of time	**The wait for saying goodbye and donation is painfully slow**<br>- I need a faster pace (taking the next step in the donation process)<br>- I do not want to wait long(er) for donation to take place<br>- I do not want to wait to say goodbye for donation<br><br>**I experience the donor conversation as an process under a high-pressure**<br>- I do not feel like I have all the time, I feel a certain pressure<br>- I must make a donation decision now (with some pressure) and pursue that choice<br><br>**I need more processing time, the donation process demands a lot from me**<br>- I need more time to process the bad news<br>- I need more processing time (in general), it is too much<br>- I am shocked by the subject of donation and want some time to think about it<br>- A lot happens after the donor conversation (a lot is coming your way)

5) Procedural clarity	**I want clarity about what I can expect regarding my relative's death**<br>- I want to know from the clinicians what is going on (the situation)<br>- I want clarity about the bad news (no false hope)<br>- I want to know exactly how saying goodbye goes WITHOUT a donation procedure<br>- I am worried about what to do next regarding death (which actions I have to take and what I have to arrange)<br><br>**I want clarity about what I can expect in the donation process**<br>- I am ignorant of the donation procedure and not well prepared<br>- I do not know what my relative's donation wish is<br>- I want clarity about and a time indication of the donation procedures<br>- I want clarity about saying goodbye and death WITH a donation procedure<br>- I want to maintain control over the situation, the donor conversation and the donation procedure<br><br>**I have a practical concern in the donor conversation**<br><br>**After the donation procedure, I have questions about the donation process and the cause/lead-up to my relative's death**
6) Involving (non-)present family	**I am concerned about my (other) family members**<br>- I care for and protect other family members (such as my children, sister, grandmother, mother)<br>- I am concerned about the possibilities of my other family members to say goodbye to our relative<br>- (in retrospect) I am cautious in the donor conversation because I feel uneasy about my other family members<br><br>**Mutual alignment and contact between family members is a necessary aspect for me in the donation process**<br>- I am frustrated that the entire family is not allowed to attend the donor conversation (due to corona)<br>- I need to align and discuss with other family members about our relative's death<br>- I need to align and discuss with other family members about the donation decision and the procedures
Other[b]	**I want good contact (humanity and clarity) with healthcare professionals**<br>- I am concerned about moments where clinicians treat me unpleasantly<br>- The organisation of care and healthcare professionals (who are present) bothers me<br>- I feel uncomfortable and insecure about my own attitude towards the clinician(s) during the donor conversation(s)

[a](Sub)Categories were formulated from the perspective of the relative(s) of the patient and indicated as concerns.

[b]These concerns were not reported in the result section as these were ‘general’ concerns that also could apply for patient/family care in general.
*The “I” refers to the relative(s) of the patient that was present in the donor conversation and/or the supplementary interview.
