# Supplementary material for: Family concerns in organ donor conversations: a qualitative embedded multiple-case study
Source: Crit Care. 2024 Dec 27;28:434. doi: 10.1186/s13054-024-05198-2 (PMC11673370; doi:10.1186/s13054-024-05198-2)
Supplement: Supplementary file 6 — Additional file6 (PDF 338 kb) [file 13054_2024_5198_MOESM6_ESM.pdf]

Manuscript title:

Family concerns in organ donor conversations: a qualitative embedded multiple-case study

Corresponding author: Gert Olthuis, gert.olthuis@radboudumc.nl

SUPPLEMENTARY INFORMATION

Additional file 6. Illustrative quotations.

Q no.	ID number	Quote
1	F11Case010	I could kind of switch that [the emotions regarding patient's impending death] off, because I thought: "I should make sure now that I ask my own questions." As I said, that helps me if I just – that I feel like I've asked for everything I could. (..) Sure, there are a lot of emotions present at that moment. But I tried for myself to also try to leave a bot of ratio on, because I don't want to have the feeling afterwards "I wish I had done that differently…..". (..) Everyone does it [coping with patient's loss] differently, also the grieving process now. (..) And sometimes your sadness suddenly comes out very hard and sometimes you try to put it away for a while.. for the greater good or as far as there is something good. And so I think that's what I did in that conversation [about donation] as well. That I was trying to.. just for a second… I don't know… Can you turn of your emotions? Don't know. But at least I tried to focus on what I wanted to know. And I think that is also a way of showing your emotions… just by rattling and asking questions.
2	F21_F22Case019	Partner: I didn't feel the need [for information] at that time [the donor conversation] either. You have so much shit on that day, let's just say, then I wouldn't be interested in how and what exactly happens, because- But, that's for me personal, let me put it that way.<br>Mother-in-law: Yes, I had that too. I mean we were so high in our emotions, and especially with those kids also.
	F27_F32Case024	Mother: You are then [at the moment of the donor conversation] so full of your emotions, that you.. I can't remember much anymore. (..) At that moment, you mainly listened [to the clinician]. I actually memorised it very badly. (..) Just like having a conversation and then just listening quietly and that you're flabbergasted and you think like: "what… is this about our child?" (..) I remember very little of the conversation. (..) For example, I wanted to know if she [the patient] still responded when she came in to the emergency room or questions like that. (..) But that are questions you don't ask when you're sitting there [in the donor conversation], because you're just amased. (..) We've tried together to… "businesslike", I think is the wrong word, but as straight forward as possible… we've tried to think carefully about whether we're making the right decision and all that. (..) The emotions were completely turned off. Of course, I have had emotions when I was with *name deceased daughther*. Then you lose yourself completely. But then you close that door again and then I go on.<br>Father: In retrospect… You undergo it [the donor conversation] to a large extent as well.<br>Mo: Yeah, I was kind of in shock. I was shaking all the time. Really, really shaking. My whole body went back and forth. (..) That really took three days or so. So you are actually quite curious if you've remembered all that [the information about donation]. I think we did a lot- That it was enough for us, what we've heard to get a picture of it [donation]. Let me put it this way. I don't know if I've remembered everything. (..) And so much happens, that you just can't remember all the details.
3	F8Case007	That was a very strong emotional reaction, that [donor conversation] was not what we wanted to be doing. (..) [If was preoccupied with] the farewell of the father of my children and my husband. Having all the time for t hat and having all the space for that, that is of course what comes first and what is the only thing that matters at that moment. I just didn't want to have the conversation about that [donation]. It was too much, it wasn't what we wanted to give our energy to at that moment. (..) You [the interviewer] keep talking about the conversation, but organ donation was only a very small topic of the conversation. The conversation was mainly about saying goodbye to *name deceased husband*. (..) I didn't had the energy to take information in about that [donation]. So actually, to put it bluntly, I don't really care what it [donation] meant for the goodbye. We wanted to say goodbye the way we wanted to do it (..), we wanted to be there… we wanted him to, yeah we didn't even wanted to think about that [saying goodbye with donation], that was too emotional, too dramatic, too pragmatic, too cognitive, everything you just don't want at that moment. You don't want to have a conversation about it. (..) I think it becomes

		clear, this topic was just not something that mattered to me in light of what happened, it just didn't matter to me, not for my kids. I just didn't match the event, so. That's actually the story.
4	F27_F32Case024	Mother: And of course, I still- I have thought about that a lot… did we make the right decision [to stop treatment]? Shouldn't we have waited months to see what our child's brain is still capable of? And I have quite a bit of trouble with that. But you [the spouse of the mother: the father] was there- you were more convinced of the doctors, weren't you?<br>Father: Yes, the doctors were also clear. And that- yes, it's hard for them too, because it's really bad news you have to bring. But they were very clear of eh- "There's really no way our of this", so to speak.<br>(..)<br>Mo: Well, at some point, of course, what I just said to you was that I thought: couldn't that brain just have started working again? Shouldn't they have just- shouldn't we have waited months more? (..) But later you will, of course, have questions that make you think yes, shouldn't we have tried more? You always want to keep your child with you. But when the doctors say that [the bad news], you trust them anyway. There were people around here who asked questions about it, like why didn't you do it [waiting]? And then you do start doubting. But then you talk about it together again and then yeah, I assume that a doctor who has sworn an oath about that, will do his utmost for that [making a patient better]. (..) Because that person also told us "my experience was that doctors also reacted very differently when it came to donation. Because then they didn't do anything [treatment] anymore." I said that I could not imagine that.<br>Fa: You could in a way- yeah, if you looked at it negatively you might start to think that those doctors are after those organs and not saving someone's life, say *daughter's name*. (..) That is also difficult. You also have to put those thoughts aside, because you can't do anything with it. You just have to- you have to blindly trust that those doctors did everything they could [for our daughter].
5	F21_F22Case019	Partner: The [donor] conversation started with: "we also have someone from donation with us [the organ donor coordinator], because she will discuss things with you, as she [his partner, the patient] is registered as a donor." (..) In my opinion, she was there like: "oh yes, we have one [a donor]." (..)<br>Mother-in-law: All the papers already ready on her lap (laughs).<br>P: Yes, everything already on her lap and ready to… but yeah (..).<br>Mo-in-law: Like: "let's fill this in (laughs)". (..)<br>P: In our view, there was no question of her being a donor [she did not want it]. I had been thinking, oh that's going to be a tug of war. Then she [the organ donor coordinator] sits there with a complete folder and, just what I said, excited, in my opinion, that they have one [donor]. There is really someone present specifically for her insides, so to speak. (..) And eh… too bad for her, or at least… of course you draw your own plan, but yeah, you do feel accused, or burdened to say "we know that she did not registered explicitly", because there is a specific person present to go through with that [donation] anyway. (..)<br>Mo-in-law: (..) He [the doctor] eventually said like (..) "It's clear that she [the patient] didn't want it [donation], you're unanimous on that, he said. And then I was really like, oh yes! Really such a sign, we don't have to discuss it, they respect it, while she was really registered as a donor [with presumed consent], of course. If they had said "sorry but she really is a donor", then we wouldn't have been able to say anything in return at all.<br>P: That bothered me too, that um- that had crossed my mind too. Then I think- I know, it's been on the radio for three years [that you have to register otherwise you are automatically registered with presumed consent for donation], and in the meantime it came by once, and she [his partner, the patient] knew that too, and eh at this age you definitely don't assume you would become [a donor]. I know she didn't fill in anything [mo-in-law: me too), because the letter of November [from the government] was still closed. Completely closed. So it wasn't that she had any doubts. It's not that I slept badly because we didn't do it [donation], no, I'm just sure she didn't want it. But I thought: this will be a tug of war, she must be… they must have her organs, because she is registered as one [a donor]. (..) And when that man [the doctor] gave the conclusion of "we are not doing it, unanimously", then you have something like "Phew, fortunately". In my opinion, they also sat there for her organs.

		Mo-in-law: I was still kind of afraid that they would say: “sorry it’s registered, she’s a donor”. Not for myself, but especially for the children. I had the feeling that the doctor saved the situation this time, that’s what it really felt like. (..) That feeling [fear] was with me, because she did not [actively] had indicated that she didn’t want to be [a donor], we knew she didn’t want it, but I was afraid of a discussion [with the doctors]. That it was going to happen [donation]. (..)<br>P: (..) You think “oh god, how are we going to do this”, just the thoughts of “oh dear, oh dear, oh dear…” till he [the doctor] says no [no donation]. (..)<br>Mo-in-law: (..) You kind of feel, you just feel in your body, that’s how I experienced it, that I really think “Jeez, huh…, that donation person can go now”. (laughs). (..) A bit frightening like “oh dear, oh dear..” you know?<br>P: Does she have to be a donor? (..) It took a while before the redeeming word was given. (..) And I’ve thought it once, well fuck she is not registered, so she is now a donor. And then you get that conversation, and someone there is present for donation with that folder, then you think, okay now we are going to get it…
6	F11Case010	It felt a bit like we had to take that next step [the donor conversation]. And that makes sense, only what happened next [the donor conversation and stopping treatment followed by patient’s death] happened all so terribly fast. Even againgst the doctor’s expectations. The time after that [the donor conversation] was very short. (..) At one point the doctor steered towards “okay now it’s done with switching [family who were allowed to be with the patient to say goodbye, due to corona regulations]. Now it’s- we need to move on to the next step [the donor conversation]”. You feel a little steered in that. Which is logical, I think, because they [the doctors] have to steer, because you yourself are absolutely rudderless. But yeah, the process [stopping treatment and patient’s death] was pretty quick after that [the donor conversation]. So would I have wanted it differently? Perhaps I would have liked a little more of the feeling that I had been given all the time to say goodbye. Factually, we had all the time, but because it went all so quickly afterwards- (..) “that donor conversation has to be done, so now we have to make decisions.” (..) There was no room for that [to inidicate ourselves when we were ready for the donor conversations], because it had been decided for us “now you have switched enough and now we have to have the next conversation”. So, I think I would have preferred to be a little more in charge there.
7	F33_F34Case029	Mother: That’s what I mean by “we needed the time”, we were under the assumption that when we agreed to organ donation, then tissue donation would be out of the question. So, we parked that, we didn’t think about that anymore. And then in the evening, that [tissue donation] came up, in the conversation about what we wanted to donate exactly, and then you have to decide immediately. If we had had more time for that, then we might have made different choices, but now we said no [no tissue donation].<br>Father: You also need time to let it all sink in. (..) Because of course, the entire day, we went from one conversation to the other, and you also need your rest now and then.
8	F14_F15Case013	Father: Because that is what we remember most about the whole donation, that it took so long. We weren’t quite prepared for that, despite the fact that it must have been told. But that it really took from 11 AM to 10 PM, that we sat beside her [their daughter, the patient], that was a very long time for us. (..) Again, if we had known it would have taken this long, we would have said pull the plug and as soon as possible…<br>(..) We just wanted to know more. Because now I finally found out that the organs will end up in 8, 9 countries in Europe. And I didn’t know that before (Mother: me neither).<br>(..) That has been told [that DBD was going to be the donation method, instead of DCD], but we never understood that. We didn’t understand that at that time.
9	F33_F34Case029	Mother: For me, knowing more is more of a reassurance. Because then, I don’t have to make things up in my own mind and I don’t have to gamble (..) The hours were so long there [at the ICU], and I understand that you don’t want to take away hope by giving information too early that may not be relevant at all. But we wouldn’t have minded if we had some information [about donation] sooner. (..) More grounded information, because as I say the internet is full of ghost stories. You don’t know where to look, and you don’t want to look either because of those ghost stories, but you have so much time.. (..) I think it

		also suits us to be pragmatic and to want a lot of information. Other people may say “don’t give too much information, because I can’t do anything with that. (..) But for me, information is reassuring. (..) we felt the need to prepare ourselves. (..) The waiting takes a long time and you are hungry for more information.
10	F33_F34Case029	Mother: The waiting [for the donation] takes a long time and you are hungry for more information and then when it is said that at ten o’clock a doctor will come and he will come at ten past ten… Well those ten minutes take about three hours, so to speak. It’s an tensive time where you’re actually impatient to hear more, while on the other hand you’re also-<br>Father: Can’t take me long enough.<br>M: Can’t take long enough indeed, but the waiting time is long, you experience it as long. We got into the ICU on Wednesday nigh and we left Saturday night – we did go home at nights- but it feels like we were there for three weeks. (F: Yes.) (..) Because it’s very intense. You experience a lot of emotions, so in that sense, the time there is just a very long time, and you are powerless. You can’t do anything. (..) There’s nothing you can do to make it better, there’s nothing you can do (..) waiting just takes a long time and you can’t do anything. You are a bit powerless and for us she was our child, and you want to take care of your child, and you can’t. You have to leave that to others and everyone [of the professionals] who are there. You want to take care of your loved one, and you can’t, you have to leave it to others.
11	Case015<br><br><br>F17Case015	Daughter: (..) I think her [her mother’s, the patient] approach was that if she could maybe help other people with her organs, she wouldn’t mind, would she?<br>Mother: Yeah, I think so too. I think so indeed.<br><br>In the end, I actually made the decision [about donation] in consultation with my grandmother, but my grandmother was more of “you do what you want”, so in the end I also made the decision. (..) Consultation with my boyfriend was not really the case, I mean, it was more between me and my grandmother. I did ask my grandmother how she stood in the situation. Look, if my grandma had firmly said “I don’t want this [donation]”, then yeah I don’t know. I just asked her “Do you want this?” And then I explained to her about “it entails this and this and that” and then it was also “what do you think Mum would have wanted?” I said to her: “She has [a donor registration of] presumed consent, but I’m pretty sure [about her wish], because that’s [the presumed consent registration] because she didn’t return all the letters, so we did talk about it and then she [my mother] said ‘yes, I think it would be nice to do that [donate]’.” But if my grandma had said: “well, I’m really against it [donation], and presumed consent or not, that doesn’t matter to me”, that would have been a difficult situation for me, because in the end my loyalty is to my grandma at that moment and of course not to the people you can help with an organ donation. So that would have been a very difficult factor for me, if she had said “No, I really don’t want it”.
12	 <br><br>Case029<br><br><br><br><br><br>F33_F34Case029	**[Note: in this fragment, the concern is clearly articulated. We show it here as it is a nice example how concerns about other family members can be experienced by relatives. However, most oftenly these concerns were less clearly articulated at the time of the donor conversation.]**<br><br>Mother: (..) [we had] also a lot of care for *name patient’s boyfriend* who also has to cope and move on with it. Just dating and then this happens. You know, the parents of *name patient’s boyfriend* who find her [the patient] here instead of on their couch; it is a different introduction than you had hoped. (..) And you are also so afraid that those young people will take it over [commiting suicide] or copy it or that it is a nudge towards it or… and *name patient’s boyfriend* has to cope with this and move on with it, so it feels so good that he gets all the important- yeah all the support, that he ís reaching the age of 80 eventually.<br><br>Mother: We thought it was important that he *name patient’s boyfriend* could understand it all too, but we were supported on all sides [by the hospital], because when we had a conversation [with the doctors], he [patient’s boyfriend] and his parents were very realistic in that. They said: “conversations where you think it’s fine if we’re also there, then that’s good, but if you prefer to do it alone, that’s fine too. For all the conversations we did alone, the person

		[doctor] who did the conversation then took time to inform *name patient's boyfriend* and his parents. So it was very nicely facilitated by the hospital. (..) That takes a lot of care away from us. (..) But it is your daughter's boyfriend and in one way or another, if your child plays soccer and kicks in a window, you as a parent also feel responsible. So eh vicarious responsibility, that's what you feel then. And that's what we had then too. My child is dying, so we are a little responsible for the people who love her. Something like that.
13	Case013	**Several passages selected throughout the donor conversation, line numbers are indicated:**<br>**L32** Mother: This just can't be true. She [daughter] had everything on track. She has fought so hard all her life for her education, for everything. And now this.. This just can't be true. (Cries) (.. ..) This can't be… It's just not fair. I.. I can't do without *name daughter*.<br>**L50** M: I can't live without [her]. I just really can't live without her. That just doesn't work! (Cries) (.. ..) I can't … (..) This shouldn't be possible. (Cries) (..) (.. .. ..) It is just not fair. Why? (..) how can we continue living without *name daughter*? That is not possible. I cannot do it. I really can't (..)<br>**L62** M: (Cries) (.. ..) But.. How am I supposed to call *name son* that his sister will soon be gone? That's impossible. (Cries).<br>**L88** M: (Whispering voice) We've lost her. (Cries)<br>**L126** M: (Cries) But I can't live without her.<br>**L168** M: But just that I'll just never be able to.. (Cries) talk to her. (Cries) (..) That she's just gone. That's just not possible.<br>**L352** M: I still can't… seriously I can't do it. (Cries) Just that we won't see her again..<br>**L516** M: No. I just can't imagine her just being gone tomorrow. (Cries) And that I can never talk to her again. I.. I… It's so unfair. She's such a sweet girl. And she has always had to fight so hard all her life for where she is now. It's gone. It's really so unfair. If only I could do something…<br>**L724** M: It's just impossible to get hold of. It's just… just that she'll not be around us any more soon. (..) it's just… it will never be the same again.<br>**L822** M: (Cries, whispering voice) I just can't get it.
14	Case010	Partner: But will he [the patient] die with you [clinician] on the operation table?<br>Clinician: (..)<br>Daughter: Well, I want one thing and that is for daddy to breathe his last breath with us.<br>P: Yes, I want that too.<br>D: That to me is really… **very** important.
15	Case014	Daughter: And how long would this [the donation procedure] take? You just said that it might take a while… but-<br>Clinician: It may take a while. It's always an estimate for me whether that's one day, or whether it's 1.5 days (D: okay). For example, Sunday morning could be the moment that we say "we are going to stop the treatment" or we see that brain death has been diagnosed at that moment. Then, we schedule a time when she [the relative] goes into the operating room.<br>D: Okay.
16	Case008	**Not explicitly communication to postpose a response**<br>*The clinician does not respond immediately to the family concern(s). Later in the conversation, the clinician herself returns to the aforementioned concern.*<br>**L200** Partner: But I know my son is not ready yet [to accept patient's death]. And my daughter, because of the operation she underwent, she is, of course, even more emotional than usual. **[no reaction of clinician]**<br>**L412** P: And it will be difficult. My son isn't that far yet. I called with him, because I thought he should also know my stance in the situation [to stop treatment]. (..) He saw miracles happen. And then I say to him: "Son, those miracles are not coming". I mean, I'm not a doctor, but the situation here… was clear to me. **[no reaction of clinician]**<br>**[Later in the conversation]**

		**L680** Clinician: And with your youngest son? He is not that far yet, you say. That realisation or… You have another daughter who is still in the hospital [because of the operation], and then your son…
17	Case026	**[After telling the partner about the donor registration and the donation procedures]**<br>Clinician: What do you think if I discuss all this [donation procedures] with you?<br>Partner: **Heavy**. Look, *name relative* was a very social person, so I think he would have wanted it [donation].<br>Sister: I think so too. But after four hours [after patient's death], it is no longer possible to donate? **[more practical concern]**<br>C: No, two hours is the limit and it also differs per organ. **[Clinician responds to the practical question of the sister by explaining the donation procedures elaborately. He does not ask what the partner means with "heavy", he does not probe this cue. In that way he does not respond to the partner's experienced burden of the potential donation, which is indicated with the cue "heavy"]**<br>(..)<br>P: (..) Just the donation… I was like **oh oh!** You know? But yeah, I just know he would have wanted-<br>C: Yeah okay.<br>P: …And that it's very important that it is done.<br>C: It is a very noble thing indeed, but it also can provide an extra emotional charge and tension. We have to be honest about that too. **[Here, the clinician does mention a psychological burden of donation, but does not explore and probes what the partner actually experiences as a burden emotionally]**<br>P: That's true. (..) I'm pretty level-headed about it, but at first instance you do have something like **ah yuk… oeh! It's [donation] just not a pleasant idea.** Yes, but it is very important and I agree with that. **[Here, the partner again mentions here emotional concern about donation. Again the clinicians does not probe what exactly bothers her about it.]**<br>Sister: But if I understand it correctly, organ transplants are pretty… when I hear people tell me how fast you have to transplant [the organs]. Then I also understand those waiting lists and that it is really an issue.<br>C: Yes, that's right. And it also differs very much per organ…the details. **[The clinician proceeds with information about donation per organ and explains the follow-up arrangements regarding the donation.]**
18	Case008	Partner: If at some point I can't handle it [donation] anymore?<br>Clinician: You get the room over here. So…<br>P: But I can go outside for a while, for example?<br>C: Sure.<br>(..)<br>Daughter: Can we both go now [to the patient]?<br>C: Yes, you can both go there now. **[The conversation does not end and continues about follow-up organ testing for donation]**<br>(..)<br>P: I want to go outside for a moment.<br>C: Yes. **[The conversation does not end and continues about the follow-up interview for the present research study]**<br>(..)<br>P: Pff. I want fresh air.<br>Nurse: Yes.<br>C: Yes, I understand. I also told you a lot of things… I had to tell you a lot.<br>Nurse: It's a lot of information, isn't it?

		D: Yes.<br>C: That’s also important.<br>Nurse: Let it sink in quietly.<br>C: Yes.<br>Nurse: and if any questions come up later, feel free to come and see us. We will be-<br>P: I’m going to get some fresh air first.<br>Nurse: Sure. **[The conversation does not end and continues about the corona restrictions and procedures in saying goodbye to the patient]**<br>(..)<br>P: I suggest that I immediately go outside first.<br>Nurse: That’s good.<br>P: And then I will go to dad [her partner, the patient].<br>D: Then I will go with you. Let’s do that.<br>P: I just can’t *(cries)*. I can’t at the moment.<br>C: No.<br>Nurse: No, I get that.<br>D: That’s okay too. That’s allowed.<br>C: That you-<br>Nurse: Just let it sink in for a little while.<br>C: You are going outside, and- **[The conversation does not end and continues about the arrangements and planning for the donation. The partner is also asked questions about the son and daughter]**<br>(..)<br>P: I want to go outside for a moment.<br>C: Yes.<br>D: Yes, I know. Come on.<br>C: It’s enough, right? **[The conversation does not end and continues about the entire process in the hospital, the follow-up arrangements and planning for the donation, and tissue donation is named for the first time]**<br>(..)<br>P: I’m going outside now.<br>D: Let’s go outside for a while.<br>**The conversations ends.**
19	F9Case008	**In fragment 6a, the partner mentioned her youngest daughter and son, but she did not explicitly mention that she wanted to discuss the donation decision with them, for the reason that she had difficulties with decision-making and processing the information. The partner also mentioned briefly her youngest daughter’s gallbladder operation and the difficulties of her son to cope with patient’s impending death. However, she was unable to explicitly state that she was preoccupied and burdened with these thoughts [and still was in the follow-up interview], and therefore unable to process all the information about donation. She was only capable of expressing their immediate needs “do go outside”, without full reasoning. In the follow-up interview, she elaborates on her concerns, that partly still exist:**

		It is a very drastic event when you make such a decision [for donation] and in my opinion the time between being told that they [the doctors] could no longer help my husband and immediately on top of that that the donation came into the picture, that was too intense for me. (..) They could have told me: “we would like to talk to you about a few things”, and then they could mentioned that it was about donation, but maybe they could leave 1 or 1.5 hours in between. So that I could just discuss it with my children in those hours. My eldest daughter was with me, my youngest daughter just had a surgery the day before. She did come to the hospital in the evening, but she had had a gallbladder operation, which is also quite intense, so everything happened at the same time **[concerns about her youngest daughter]** and my son also came to the hospital in the evening. Anyway, then [with more time between the conversations] I could have picked up my phone outside, called her, called my son, and yeah… and the adrenaline also rushed through my daughter’s body of course [because of the operation and circumstances]. (..) The doctor said that permission [for donation] was needed from all three children. Well, then, no matter how sad you are, you should have half an hour to discuss it with them, and it actually went tak-tak-tak. “Have you thought about it, how do you feel about it?” And for example, when two kids said “we don’t want it to happen”, I would probably have said “No”, and that time wasn’t there.<br><br>**Concerns about her son:**<br>He also had quite a few conflicts with his father [the patient] lately and he was unable to talk those conflicts out with his father. So yeah, that also struck me. We all knew the cause of those conflicts, but he [son] did not want to discuss it. But it has to be discussed with him [son] again, but I will wait with that for maybe 2 to 3 months. Everything must have settled down first and then I will see if I can talk to him about it. He always said that his father was not proud of him, while his father was very proud of him.<br><br>**Concerns about her youngest daughter:**<br>I was also left and burdened by the fact that my daughter had a gallbladder surgery on Monday evening. I mean, I was also thinking about her too! It was an 2-hour operation after all and she was discharged from the hospital on Tuesday morning and on Tuesday evening she was in *name hospital* [for her father, the patient]. You normally don’t do that. Normally, you come home and then you lay down in your bed, to take a rest. But she also wanted to see her father.<br><br>**Concerns about being unable to process all the information:**<br>Interviewer: How was that [the time for processing all the information] in the donor conversation itself?<br>Partner: Well, it pretty much passed by me. I know that the doctors said that all three children had to say “Yes” to [donation]. But I was more busy with my thoughts “now *name deceased partner* is really dying and I will soon be on my own”. And I am not someone who bursts into tears somewhere, but my throat really closes and then I have a huge need to just come to myself in the fresh air. And there was no space for that.
20	F29Case026	**[In the follow-up interview, probing questions were used. The partner elaborates on her concerns, underlying her expressions in fragment 5A. In this fragment, the partner experienced troubles saying her concerns out loud and she started crying.]**<br><br>Partner: I remember that the doctor brought that [donation] up. And that it was all happening so fast, while you actually know that something like that might be happening. But then initially you have something like “No, please not!” *(laughs)*. Anyway, you get used to it [donation], of course, because you know that *name of partner, the patient* would have wanted it [donation] also. But you do not take it into account initially. You are not thinking about that. So then at a certain point when such a doctor- when you discuss it with each other, that the treatment actually no longer makes any sense, and then he starts talking about it [donation], then you really are like “Oh dear!”. *(laughs)*<br>Interviewer: and where exactly does that “Oh dear!” come from? Can you explain that a little more?

		P: Yes, that's hard to say. The idea that eh eh *(crying, followed by a silence),* that they will then eh cut in him and so on. Just wait a minute. *(cries and laughs at the same time).*<br>Interviewer: Yes, take your time. It's a shame that I'm not with you right now. *(Silence)* **[the interview was via telephone]**<br>P: That's such a- it's so hard at that moment. Like "Oh god! Oh and they're going to cut him open too…" you know. (*Laughs and cries)*(..)<br>Interviewer: That's the disadvantage that we can't see each other now of course, but I can hear a lot from your voice. I hear you are affected by this.<br>P: Yes. *(Cries).*<br>(..)<br>P: It's the feeling of- Look, they are going to open him and do it [remove organs]. And of course, you <u>know</u> that it's very useful, but <u>emotionally</u> you are like… that a difficult part, yes.
21	F13Case012	*name clinician* that was such an incredibly warm, cordial man. I don't think I could have been better off with such a person. (..) He handled everything softly and warmly so yes, I must say that I experienced that very positively. (..) Besides all the procedures he explained, we also had a lot of questions for him (..). I feel like I'm not doing it justice how I describe it now, because I was really very positive about him and about *name ICU nurse* too. (..) *name clinician* approached everything very carefully and with caution: "you can basically just tell us anything and ask anything and whatever".
22	Case014	Clinicians: Do you have questions at the moment? There will probably be many more questions yet to come, but-
23	Case021	Clinician: It [donation] has major consequences for you [the family]; we're both going to put a lot of time into it. It is an emotional burden for you, because we must perform many investigations in the next 24 hours, which is why we have to wait with stopping the treatment. We're going to make sure she's [the patient] completely comfortable in the meantime, so the pain medication continues, the sleep meds continue and we increase them if necessary. So, she doesn't notice all of that [the investigations] herself and she's in absolutely no pain.<br>Son: Yes, that is indeed important to me.<br>C:That's the most important thing.
24	Case011	**Explicitly communicating to postpone a response**<br>*The clinicians explains to the family that he will not respond to the family concern immediately, but will return to it later*.<br>Partner: And then what… Once that's all out [of his body][the organs for donation], what's going to happen inside [him]?<br>Daughter: Nothing, they fill it up neatly, you can't see anything about it.<br>Clinician: **I will tell you something about that Mrs. in a moment.** For the part, we just talked about [time schedule], we'll agree on a time (..).<br>**[Somewhat later in the conversation]**<br>C: You said: "what does that [the donation] look like afterwards? (P: yes). That's like a surgical wound. It is neatly closed again and if he… the undertaker ensures that it is neatly dressed. You don't see any of that. That's the piece around his belly. If he also donate his lungs, it would also be the part of the chest. But because he's dressed, you don't see anything of that. So that's indeed what your daughter said earlier.
25	Case023	Daughter: How long does *name brother* [also hospitalised, because he and his father were in the same accident] have to stay here? Because that's a thing…<br>Clinician: What do you say?<br>Partner: Well, my son… he is…<br>D: My little brother.<br>P: Well, he just had surgery again.<br>D: And it would be a bit silly if he couldn't be at the funeral [of his dad], of course.

		C: Yes, but that's something to look at later. The funeral is not yet there at the moment, that will be sometime… well, the second half of next week. So, we cannot steer the process on that (P and D: no, we get that). So that is something, which has to crystallise out in the second instance of how to do that, whether or not he can be there. (..) That's something you can discuss with the department next week, I think. (P: okay). Other points of interest or questions?<br>P and D: No.<br>ICU nurse: I'm thinking out loud myself, because I don't know if social work is involved, but with that kind of things, social work would sometimes also be very practical. They can help think what could be possible.<br>I: (..) That it [the concern] will be taken up.<br>(..)<br>ICU nurse: I will call *department name* [of the brother/son] later and I'll explain the situation. Then I will pass the matter on to them.<br>D and P: Thank you.
26	Case028	**L103** Partner: So, this [donation] could take a while longer than only today?<br>Clinician: Yes, this can definitely take a while. So indeed longer than today.<br>P: Oh, that too…<br>C: Yes. What I already said, it can take 24, 48 and sometimes even 72 hours. **[C provides information on organ testing and time period of procedures]**<br>**L119** P: Well, days… I think- I hope not, I must say.<br>C: No, I hope not too and we do our very best to limit everything as much as possible, but we don't always have that very well under control, do we? But we need a little time to give him that chance [to make donation possible]. That's at least one day, probably a day and a half or two, and sometimes three days. But hey, we do our utmost to keep in touch, to always be available for any questions, together with the transplant coordinators. And to try to guide you as best as possible and take you along in the process.<br>**L133** P: Well. Well, I think it's quite a burden..<br>Son: Yes, I think so too.<br>**L149** P: No we understand that very well [stopping treatment], as sad as it is. But that it can still take days from now on, that he is laying there like that…<br>C: Yes, I can give a word of explanation about that… **[clinician gives motivation for the time period of procedures, such as the quality of the organs].**<br>**L169** P: Because we come all the way from *name town*. It is also not the case that we can say "okay, I am going home for a while and come back later". That's why I find it a bit difficult.<br>C: There are also family rooms here. (..) Nothing is necessary, but just know that there are many things possible and many things that we can also offer you to try to support you.<br>**L198 [Clinician explains the concept of brain death and the time procedures of donation in case of brain death. He also provides information about the logistics regarding organ testing, allocation, transplantation and the availability of an organ donor coordinator]**<br>P: That [time period of procedures] surprises me and I find that the most difficult. (C: I get that). That the process slows down, despite that we know he is going to die. And now we have to- and you're waiting days now for that to actually happen, right?<br>C: But we can only say that's what he would wanted, of course, isn't it?<br>P: Yes, of course, I do understand that. But yeah…. Well…<br>C: How we look at it, is that we have to do it together, right? And that's why we have-<br>P: No, but it overwhelms me. I didn't really know how it all worked. Fortunately… (..) but, yes, I think this is peculiar.<br>S: Look, I understood that it is a few hours longer.

		P: Yes, I would have thought so, but that it could take days for him to lay like this.. If I'm honest, I'm not going to sit here for days… (I: no, but it won't last…) I can't take that either.<br>C: That's what I'm saying. It doesn't always take days.<br>P: We haven't slept all night.<br>C: It's possible… it rarely takes less than 24 hours. But it also rarely lasts more than a few days. But, it doesn't take just 1.5 hours. We can tell you that.<br>**[Clinician elaborates on time procedures]**<br>P: It's a great story from your side, of course, but it surprises me and I think you too (S: yes.). Anyway, the choice has been made, so we have to go along with it. Then we'll just sit with him for a while now and then we'll just go home for a while I guess.<br>S: Yes, I think so too. (..)<br>C: The most important thing I want to say is… that a lot is possible. And there's- I think almost everything is possible, but just try to stay in touch (P: hmhm), we'll see what's feasible and how we can help you. That's a- It's a tough time and I think we as doctors should express our gratitude from society as well, right? That people want to donate, and that is to his credit and to you as well.<br>P: Well, that's not my problem either. It's just that this overtakes me: that this is going to take <u>a long time</u>!
27	Case015	**The following passage is selected throughout the donor conversation, line numbers are indicated:**<br>**L75** Daughter: Who loses their mother at the age of 25, that's impossible, really.<br>**L141** D: *(Cries)* I just find it really bizarre. I was just writing my thesis yesterday and now I have a dead mother. Really, I can't get my head around it, just really, fucking bizarre. Sorry, I know it's not your [the clinician] fault of course, but it's just really bizarre. *(Cries)*<br>**L213** D: I don't know if I can handle it grandma or not… *(Cries)*<br>**L225** D: *(Cries)* Thank you for the explanation. And for everything you've done. I just can't believe it, my mother and my grandmother are the only family I have. It is just… (..) I just can't believe it grandma. *(Cries)*<br>**L287** D: Grandma, I can't do this, I'm just 25. *(Cries)*<br>**L279** D: Mama said to me, I'm going to spoil my grandchildren. Now my grandchildren are just never going to know a grandma. That shouldn't be possible, right? When I get married, I just don't have any family around. That can't be true. If I become a lawyer, who will sit in court with me?<br>(..) I'm only 25, This is not possible. *(Cries)*<br>**L329** D: Oh my god, grandma. *(Cries)* Now, just at my age of 25, I'm an orphan. That can't be true. This isn't fair.
28	Case009	Partner: (..) But what [is the plan] now? Are you going to stop [the treatment] immediately? **[Concern about timing]**<br>Clinician: Yes, I think we should talk about that. Anyway, you should have plenty of time to say goodbye in a way that is pleasant for you. That is the most important thing and that is paramount, because you can only do that once.<br>P: And others [family members] are not allowed right? **[Concern about timing is substituted by concern about non-present family]**<br>**[The concern about timing reappears later]**<br>P: But then, what is the procedure? Then, of course, he must be kept alive for the time being, so to speak.<br>**[Clinicians explains donation procedures]**<br>**[Somewhat later the concern about timing is presented again]**<br>P: And is that [donation] still happening today, or…?<br>C: Yes, but the only condition… here we go… the only condition for that is that we are really sure he is brain death. So we clamp those drains, so that the pressure in his head increases again and then he becomes brain dead, because up till now he is not brain death. That's a way that we think would be best for organ donation. The best organs…

		P: I think it sounds very awful. (..) It's like you're deliberately killing him for his organs. To put it bluntly. **[Concern about timing is again substituted by another concern; fear about donation]**
29	Case011	**Example 1**<br>Clinician: Madam, we will take care of it, anyway, we will talk about that later with your GP, that you don't have to worry about yourself now. (..) it will all be different [your life at home] and not as you had in mind et cetera, but there is no acute problem. We can make sure that you know that together. We can take care of that together, just so you know.<br>(..)<br>Partner: *(Crying)* Washing, he did everything for me… the laundry… het did everything.<br>C: Do you know ma'am, that can be solved practically. (..)<br>(..)<br>C: Let us know what you think we can help you with. We can arrange the ambulance and at home, the GP must also help with that, but if we can facilitate something in this, please let us know.<br><br>**Example 2**<br>Daughter: Okay, to the point, because I just have a splitting headache. What are we going to do now, next step. *(she summarises the arrangements and future steps]*. Maybe very briefly, but I didn't sleep all night, I was already tired yesterday and I didn't sleep all night. This is all to much for me, so I'm like: pfff, I want to take a shower, change clothes. Blegh.<br>C: You can also take a shower with us, if you want. We just have a shower, so we can just arrange that. (D: laughs).<br>(..)<br>If we can do something for you, that shower or something else, please let us know or something to drink or whatever, we will be happy to help you with that. That's what we can do.
30	Case009	Partner: It's like you're deliberately killing him for his organs [with donation]. To put it bluntly. **[This concern is based on lacking information: the patient is not killed for his organs.]**<br>Clinician: The other way [of donation] is that we take the ventilator off. That we don't wait for brain death to occur, that we let him die, here, take the respirator off and then he'll be dead in no time (..). **[the clinicians describes the procedures surrounding donation after brain death and circulatory death, but does not explicitly provide the information that the patient is not killed for his organs. In that sense, the response does not fit with the needs of the partner. The expressed fear is not resolved or softened]**<br>(..)<br>P: I've always said: as long as they don't declare him dead sooner [with donation] or something (laughs) … I think that trauma is still there from the past. **[concern reappears]**<br>C: No, but it's more about you. You have to continue with this, you continue living, you go on. And you're going to have memories. (..) You must do what he would have wanted, but it must also feel good for you. **[the clinician again gives some information, but does not align with partner's needs. The expressed fear is not resolved or softened]**<br>P: Yes, but as I already said: I can't imagine how it would be… what I said: I think it sounds awful. **[concern reappears]**<br>C: I get that, that's difficult… but that's also… that a technical story, but eh… what's going to happen… (further technical information). **[Again the clinicians provides information; the technical story about the procedures, but does not address explicitly that the patient is not killed for his organs. The expressed fear still remains].**

31	F28Case025	Then I thought, come on with the data [about donation] *(laughs)*. (..) Like tjak tjak tjak, this is going to happen. (..) not everyone is like me (..) that's my only, I can't call it a criticism, a complaint, but the only thing is that I wanted a little more pace, you know. It [donation] was actually massaged in like that, and of course that's good. But look we already saw her for 12 days [in the hospital] (..) Another person can sit with a potential donor who was brought in by a traffic accident. Well, that's the first shock: that someone can't get better. Then there's a whole different approach, and in that cases that approach [of slowing down] will probably be fine *(laughs).* (..) It felt like he told it [information about donation] in slow motion. *(laughs)* (..) I'm more like, we we already said yes [to donation], and now, let's do it, done.
32	F18Case016	Partner: Then [after receiving the bad news] I told the clinician to talk about donation. Then he said: "Yes, but… we'll will first give you some time." I was a bit too early with donation I guess, (..). I may have gone a step too far, but that was because I already knew some things about donation. (..) I noticed that in the conversation I went a little too fast, that they [the clinicians] were still making a run-up towards the topic. But I didn't need that run-up, let me just jump right away. (..) For me, that [wanting to know all the details of the procedure] is not very important. (..) It [the information] doesn't mean much to me anymore, my loved one was no longer there, in my opinion he was actually already moving to a different form of being.
33	Case008	Partner: I've already indicated this week, but I don't know how that goes these days… I have experienced once, that the family was told: "we are stopping the treatment." And that they [the clinicians] pulled the plug… on the spot, in front of the family..<br>Clinician: No, we don't do that. No.<br>P: No, but do you get that? If you've experienced that once, it's very scary to see.<br>C: Yeah, that's indeed very scary.
34	Case011	Son: Couldn't that be faster?<br>Clinician: That he dies? I expect him to pass away soon but…<br>S: Because eh… for the reason that…<br>Daughter: You have to let him do it himself. They can't commit euthanasia or anything.<br>S: No.. okay.<br>I: I see.. I think… I have an idea of your **concern** and why you ask it, but we can't… the goal shouldn't be that he dies quickly, that is not allowed, what is allowed is that we make sure that one does not suffer (..).
35	F1Case001	For myself, it was, of course, quite emotional: a lot is changing, a lot is uncertain. You balance between hope and sadness, and questions, and everything is actually very uncertain, very unclear. And yes, you have- you actually kind of lose control of the situation. (..) Well, unclear in the sense of a major situation. You knew that the situation was very serious, but the unclarity or the uncertainty of which way it will go… (..) You go from hope and work to recovery in one fell swoop to saying goodbye and I was, therefore, more concerned with that, than with the organ donation. I hadn't really thought about that at that time. (..) It was actually the 2nd blow after the blow of "the treatment will be stopped". (..) You want to hold on, you don't want to let go. And both are indications that you need to let go. And you just don't want that.
36	Case004	Son: But to put it succinctly, it's now up to us to say what we are going to do with him [stopping treatment or not] or how should that be done?
37	F27_F32Case024	Mother: Then I said to one of my girls [other daughters]: (..) Hey, do you remember – "Yes, said *name daughter*, *deceased daughter's name* wanted to donate." I said yes, that's right. Will you tell that to the doctors? And that's how it really started.
	Case024	Mother: You would prefer not to do it [donation], but I have registered myself also [as donor] and *daughter's name [patient]* wanted this too. We had discussions about it so many times, so she really wanted that…<br>Father: About donation? Yes.<br>M: … Yes, so we just really <u>must</u> do it.

		Clinician: Sure, at this moment it is- yes, it feels very difficult now of course.<br>M: Yes. Of course you want… you don't want your child to be cut in…<br>C: Exactly.<br>M: But, I think, I would also be very grateful if another kid, or someone else… [is helped with the donation]<br>F: She [the patient] would get mad at us, if we didn't [donate].
38	Case017	Clinician: (..) She herself has indicated that she wants to leave the decision, whether or not she would become an organ donor, to the next of kin and then actually that would mean you as a partner and you as parents, and you as first-degree family as it is so oficially called.<br>Sister: *(sigh).* This is a tough conversation, because now I'm starting to realise it a bit, that we have to decide…. Jesusmina. *(Sign)*<br>*(several family members are emotional)*<br>Partner: Phew. I…<br>S: Yes, and you want an answer to that right now?
39	Case011	Partner: No, but I'm going home [instead of staying in the hospital till the relative's death and donation]. I want to go home. I can't stand it anymore.<br>Daughter: Then I think it would be better if you say goodbye to daddy now. (P: Yes.) And then we go home. And that… (P: No). And that when the times comes… you want to go home right?<br>P: I want to go home.<br>D: Yes, that's what I'm saying, that you now say goodbye to daddy, the way he is now. (P: Yes.) And that we just go home and then we [daughter and son] come back later to really [say goodbye], at least I want to be there. (Son: me too). That we come back for a farewell…<br>S: Then she [partner] won't have to go back.<br>P: I can't manage that anymore.<br>D: No, you don't have to.<br>**[Clinician responds by explaining that the only option to say farewell to the patient is in the hospital, not at home]**<br>P: But I can't make it [staying here]. I… *(coughs).*<br>S: Physically, she can't take it anymore, not here either [points to his head]. So uh don't [stay here].<br>**[Clinicians responds by explaining his personal interpretation of saying goodbye and it's importance. He emphasises to be at peace with the way of saying goodbye for optimal grieving]**<br>P: No, no. I'm saying goodbye now and I'm not coming back. I can't anymore. I can't anymore.<br>D: Then we'll make sure you can go home.<br>P: I really can't take it anymore. So. (..) I really can't… I can't take it anymore.
40	Case025	Stepson: Okay, but she's basically kept alive longer with that donation.<br>Clinician: In that sense… the moment you give permission for donation, I will explain that… that takes time and that is in a sense…<br>S: Okay, but isn't she suffering?<br>C: That is the most important thing at all times, that we know that her comfort is not compromised.<br>(..)<br>S: It's just important to me that she's not in pain. That just matters to me.
41	F30Case027	It is my husband, it is our father, he is no longer with us, but his body is also ours and you do not want to give that away. You want to cherish it, you want to respect it, you want to treat it well, you want to lay it down nicely, you want to say goodbye to it in your own way, you just don't want other people around it who may not treat it respectfully, who take out what they need [organs] and then discard it. Do you understand?

42	Case007	Partner: No, we’re not going to do that [donation]… We [the family] talked about it.<br>(..) I: And then I also wanted to ask you: why are you so clear about that?<br>P: Well, that’s a matter of feeling. That’s all. (..) It does not feel right. (..) That’s it.
43	Case005	Partner: We have said “yes” in principle, so to backtrack that now… It is a whole situation of course, but yeah…<br>Son: What you want… If you say “well eh….”<br>Clinician: Yes, I can’t make the situation easier, because the whole process of saving those other people… there is a lot [of procedures] involved.<br>P: Yes, well, we said yes earlier, so eh… then that should proceed… (..)<br>(..)<br>P: It is a bit more of a situation than we thought [especially the waiting period], but we have now said yes, so eh then that should happen.
44	Case014	Daughter 1: I just want to know how it is going to… *(crying, very softly)*<br>Daughter 2: You want to know what happens now?<br>D1: Yes.
	Case012	Father: But then I have one more question. What is the time of death then [with donation]?
	F33_F34Case029	Mother: I need information. When she [the clinician] starts to tell [information], a question pops up in my head. My husband, he can ten easily think ‘oh well, that answer will come shortly’. It works like this for me: when I have a question, it keeps rattling around in my head and that hinders me, it costs me so much energy that I can’t listen properly to what she tells me, so then I only hear half of the information till I have my answer. (..) Because that’s what I’m focused on, so I need to ask those questions. (..) In that sense, I can be overwhelming if I don’t give an explanation [how that works for me], but I do have seven questions while you do three things. But on the other hand, I think hello it’s MY child lying there. You are just a doctor in the service of my child, give me that information or explain to me why you can’t give the information to me.
45	Case004	Daughter-in-law: Someone else is actually coming [to the ICU]…<br>Son 1: My aunt and uncle are on their way.<br>D-i-l: Can they please be here?<br>Son 2: Can they please come here?<br>D-i-l: I know it’s not allowed but… *(Crying)*<br>S2: That’s my second mother.<br>**[ICU nurse explains pandemic restrictions]**<br>D-i-l: Isn’t it only possible here [in the family room], not to *name patient*, but just here. *(Crying)*<br>S2: That’s really…<br>D-i-l: The only thing…<br>S2: Our mother passed away 8 years ago *(crying)…* Otherwise we will go outside. She is just a bit the person who took on the mother role. *(Crying)*.<br>D-i-l: She is very caring.<br>ICU nurse: We can come back to that in a moment.<br>Clinician: Yes, we can come back to that in a moment.<br>S1: Yes, please think about it.<br>D-i-l: I know we ask a lot but…

		**[ICU nurse explains pandemic restrictions]**<br>(..)<br>S1: I don't know what time they are here.<br>C: We totally get it, we also struggle with those rules, so well… first let's just-<br>(..)<br>Aunt: What about *name niece [sister of sons]*?<br>D-i-l: We can't get hold of her.<br>C: Yes, I understood you have another sister who is in Australia that you can't reach?<br>S2: Yes, we can't get her on the phone.<br>S1: She is in meditation for 10 days.<br>A: But you have already tried that phone number?<br>D-i-l: Yes, you will receive voicemail right away.<br>**[Sons talk more about phoning their sister]**<br>S1: Because, I think practically, how long (S2: yeah how long is this [keeping patient alive] still possible…) can we stretch daddy's life, so that my sister can possibly say goodbye or how should I see that?<br>**[Clinician explains his instability and the need to avoid suffering]**<br>A: Then you should at least take pictures of him or something for *name niece*.<br>(..)<br>S1: Are people still allowed to come over?<br>S2: Are people allowed to come by at all? (C: Sure, yes). Because then I will have his brother come over and (A: yes).<br>S1: And his mother.<br>(..)<br>S1: But if family gets in the care now and comes here, they can say goodbye now? (C: sure). Okay then we're going to call people. Yes, we have to…<br>(..)<br>A: We sent a SMS message [to niece] with an urgent request to call, but I'm trying also other ways now… send an email and maybe friend who can drive there… Phew….<br>(..)<br>S2: We haven't talked about that [donation] yet, we have been calling all people and…
46	F1Case001	At one point, I thought something like: and if *name deceased partner* had donated his organs, how would that have gone? (..) I just want to [evaluate] the whole process. A scan was made and, on that basis, it was decided to stop the treatment. I would very much like to see that scan and discuss it. At that moment it was said "essential parts are affected, so this is not compatible with life", but yes when you are a while further, you still want to know more about it. I really want to discuss those things. And then indirectly also discuss the stopping of the treatment, and the questions I had about organ donation may also come up.
47	F21_F22Case019	Partner: Then, yes, at a certain point there, during such a conversation [about donation], it is of course told that she [his partner, the patient] is going to die, so the emotions are very high of course. So [in that conversation] you don't think about things such as "We have to arrange a funeral. We have to do this and we have to do that" [you find that out later]. And we actually thought that too little attention was paid to that [by the doctors].
48	Case003	Partner: I think it's important now that people are with him who… (Son: let's see who we should call later. Daughter: Yes). *(Crying)* Oh my god.

Met opmerkingen [MMvd1]: Deze wordt niet in de tekst gebruikt, alleen in figuur 1, klopt dat?

	Clinician: Step by step.<br>(..)<br>P: (..) Everything in his house is from *name patient* and that breathes *name patient*. Then, it is actually indigestible that that will change soon, because we have everything [household goods] double, and I have my house there and I have my things there. And then I will move of course [to patient’s house], but then a lot of things have to go, and quite a lot. a<br>C: You are thinking a lot [in the future].<br>S: A lot of steps forwards, isn’t it?<br>C: Just try to stay here and we’ll talk you through this.<br>P: Yes, yes, but all these days, I have been thinking about this.<br>C: I get that. There is a big job [for you] here now [saying goodbye and donation], so we are going to help you through that. First, here.<br>D: First things first.<br>P: Yes.<br>C: Good. I’m going to call for the other doctor. To discuss that [the donation] and then we will have contact later about how and what exactly.
